# The Electric Field Instrument (EFI) for the TRACERS Mission

**DOI:** 10.1007/s11214-025-01202-5

**Published:** 2025-09-09

**Authors:** J. W. Bonnell, M. Ludlam, A. Slagle, K. Goodrich, J. W. LaBelle

**Affiliations:** 1 Space Sciences Laboratory, University of California, 7 Gauss Way, Berkeley, 94720-7450 CA USA; 2 Department of Physics and Astronomy, West Virginia University, White Hall, PO Box 6315, Morgantown, 26506-6315 WV USA; 3 Department of Physics and Astronomy, Dartmouth College, Wilder Hall, Room 336, Hanover, 03755 NH USA

**Keywords:** Magnetosphere, Magnetic reconnection, Electromagnetic field, TRACERS mission, Electric field

## Abstract

The Electric Field Instrument (EFI) for the NASA Tandem Reconnection and Cusp Electrodynamics Reconnaissance Satellites (TRACERS) mission provides measurements of the electric field from DC to nearly 10 MHz on two closely-spaced spacecraft in low Earth orbit passing through the terrestrial cusp region. As measured by EFI, the plasma convection fields, ULF and ELF fluctuations, and natural HF emissions provide key measurements of plasma flow, plasma waves, and plasma density that support all three science objectives of the TRACERS mission. Here, we describe the mechanical and electrical design of the EFI, the data products it produces, and the concept of its on-orbit operations.

## Introduction

While recent missions have made significant inroads in understanding the physics of the dayside reconnection process that couples the energy and momentum of the solar wind and the interplanetary magnetic field (IMF) into the terrestrial magnetosphere (Burch and Phan 2016; Burch and Hwang 2021), the details of the spatial distribution and temporal variation of that reconnection across the dayside magnetopause remain unresolved. the NASA TRACERS mission measures the spatial and temporal dependence of that coupling on solar wind and IMF conditions through low-altitude, two-point measurements of particle precipitation, plasma flows, and electromagnetic fluctuations. Measuring those electrodynamic connections between the terrestrial magnetosphere and ionosphere requires the accurate measurement of the ambient electric field at TRACERS, and that task is fulfilled by the Electric Field Instrument (EFI) on the TRACERS spacecraft.

EFI on the TRACERS spacecraft measures the ambient electric field using the standard double probe technique (Pedersen et al. 1998; Mozer 2016). Four spherical sensors mounted on orthogonal 3-m long stacer booms provide a 7-m sensor-sensor-to-sensor separation on a spacecraft spinning at 10 RPM (6-s spin period). two of the four stacer booms form a dipole antenna with a separation of 4 meters (stacer center to stacer center) used for the 10 kHz to 10 MHz snapshot E-field measurements. Two-dimensional (2D) spin plane waveform measurements of both differential and single-ended sensor potentials cover the frequency range from DC up to 1 kHz, and one-dimensional differential measurements cover the VLF to HF band from 10 kHz to 10 MHz using a snapshot waveform capture receiver. From DC to 1 kHz individual sensor potentials ($V_{n}$, n = 1..4) and differential ($E_{mn} = V_{n}-V_{m}, m,n = [(1,2), (3,4)]$) sensor potentials are measured, while from 10 kHz to 10 MHz, only the differential potential is measured. The differential sensor potentials are used to estimate the ambient vector E-field associated with plasma flows and electromagnetic (EM) fluctuations. The individual sensor potentials are used to verify proper sensor operation, estimate the spacecraft floating potential, and measure the ambient plasma density with high (sub-spin-period) time resolution. Snapshots of the high frequency electric field fluctuations provide the data required to detect spectral features controlled by the ambient plasma and magnetic field conditions - the electron plasma and cyclotron frequencies, upper and lower hybrid frequencies - and from them derive the estimates of local plasma density required to interpret the observed EM fluctuations as has been done using similar data from satellites and sounding rockets (Bonnell et al. 1997; McAdams et al. 1999; Samara et al. 2008).

In the following, we describe the measurements that the EFI is required to make in the context of the TRACERS mission; and the detailed mechanical, electrical, and operational design of the EFI that meets those requirements.

## Instrument Requirements

The measurement requirements for TRACERS-EFI flow from the need to make accurate and quantitative estimates of the electric fields associated with plasma convection fields, and ULF, ELF and HF fluctuations to estimate plasma flows, plasma waves, and plasma density in support of the three science objectives of the TRACERS mission (Miles et al. 2025). These measurement requirements are summarized below.

- Measure the 2D perpendicular E field in the Region Of Interest (ROI) to an accuracy of better than ±3 mV/m.
- Measure 2D E-field fluctuations up to 1 kHz associated with cusp plasma waves.
- Measure the 1D E-field fluctuations at frequencies up to 5.2 MHz associated with the local electron plasma, cyclotron, and upper hybrid frequencies.

In addition to these instrument-specific measurement requirements, the TRACERS-EFI was required to comply with the general environmental (radiation, thermal, shock, vibration, acoustic), resource (mass, power), and compatibility (EMI/EMC (Electromagnetic Interference and Compatibility), DC magnetic cleanliness, and electrostatic cleanliness) requirements imposed at the mission level on TRACERS. The EFI team itself imposed the Electrostatic Cleanliness requirement upon the mission, and that specification (design, implementation, and verification) is detailed in an Appendix to this article.

## Design

The EFI is, in essence, no more than a set of high input impedance, low noise, broadband digital voltmeters. By measuring the potential difference between pairs of electrodes, conventionally called sensors, separated along orthogonal axes, one can estimate the external vector electric field (Pedersen et al. 1998; Mozer 2016).

Complications in mechanical design arise because of the need to separate the electrodes of the individual sensors by significant distances from both the spacecraft and each other. This reduces systematic errors due to the influence of the spacecraft itself, and increases the signal level for a given external field strength. Mathematically, the potential difference between two probes is given by $V_{n}-V_{m} = -\mathbf{E}\cdot (\mathbf{x}_{n}-\mathbf{x}_{m}) + \delta V_{n} - \delta V_{m}$, where $V_{n}$ and $V_{m}$ are the electric potentials of sensors $n$ and $m$ relative to spacecraft (SC) electrical ground; $\mathbf{E}$ is the external electric field; $\mathbf{x}_{n}$ and $\mathbf{x}_{m}$ are the locations of sensors $n$ and $m$; and $\delta V_{n}$ and $\delta V_{m}$ reflect any DC or AC voltage offsets in the sensor potential measurements arising from asymmetrical surface charging of the spacecraft (SC) or the sensors, and any radiated or conducted EM noise from the SC.

Complications in the electrical design arise from the need to control the electrical properties of the plasma sheaths that form around the sensors; the emission and collection of photoelectrons and collection of ambient electrons and ions by the sensors and surrounding surfaces on the booms and spacecraft; and the DC offsets and AC noise levels present on each of the sensors.

Because the TRACERS science objectives only require the measurement of $\mathbf{E_{\perp}}$, and the TRACERS mission design ensures that the spacecraft (SC) spin axis is within 15 deg of alignment to B in the nominal ROI (northern cusp) 84% of the time, and is within 25 deg alignment 97% of the time (Petrinec et al. 2025), the EFI only deploys four sensors in the spin plane of the SC. The two differential signals from those sensors, when combined with the assumption that the quasi-DC $E_{\parallel} = 0$ (i.e. $\mathbf{E} \cdot \mathbf{B} = 0$) allow for the estimation of both components of the quasi-DC Eperp with the required accuracy within the TRACERS ROI. In the worst case of a 25 deg offset between the spin axis and B, the uncertainty in the in-meridian component of $\mathbf{E{\perp}}$ increases by a factor of $1/\cos(25^{\circ}) \approx 1.1$. The on-orbit cross-calibration of the EFI data against the zero-order $\mathbf{-V \times B}$ described in Sect. 4 allows for the measurment and correction of this effect.

Outside the ROI the fixed daily alignment of the spin axis with the northern cusp B field means that in some regions of the orbit the local B field lies near or within the spin plane, which will not allow accurate estimation of both components of $\mathbf{E_{\perp}}$. Based on the current operation plan described in (Petrinec et al. 2025), the local $\mathbf{B}$ field lies within 20 deg of the spin plane (a increase in uncertainty of at least a factor of 2.9) for magnetic latitudes spanning a range of 20 to 25 deg southward of 30 to 50 deg magnetic latitude (MLAT) near the southern cusp, and spanning a range of 10 to 20 deg northward of 10 to 25 deg MLAT at northern mid-latitudes on the nightside. In these regions the data will be flagged so that users know that only one component of $\mathbf{E_{\perp}}$ is well-determined.

**Fig. 1:**
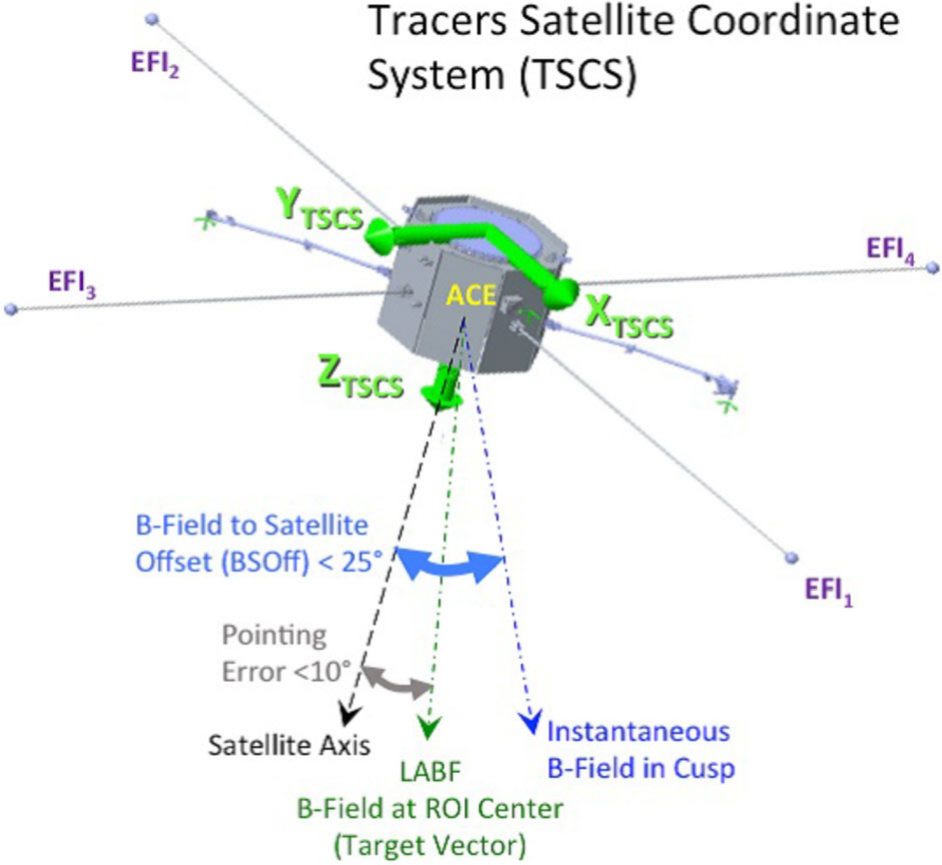
A diagram showing the deployed configuration of the EFI sensors and stacer booms relative to the TRACERS Spacecraft Coordinate System (TSCS). Note that EFI stacer boom lengths are not shown to scale in this diagram, and that the deployment mechanisms shown here for the MAG, MAGIC, and MSC sensors are not the fixed brackets used in the flight design. The TRACERS SC is roughly 1-m face-to-face and 1-m tall. Each stacer boom is 3 meters long, separating opposite EFI sensors (e.g. $\mathrm{EFI}_{1}$ and $\mathrm{EFI}_{2}$) by 7 meters. The $\mathrm{EFI}_{1}$ and $\mathrm{EFI}_{2}$ form the dipole antenna feeding the high-frequency section of the EFI instrument with two 3-m long cylindrical elements with centers separated by 4 meters

**Fig. 2:**
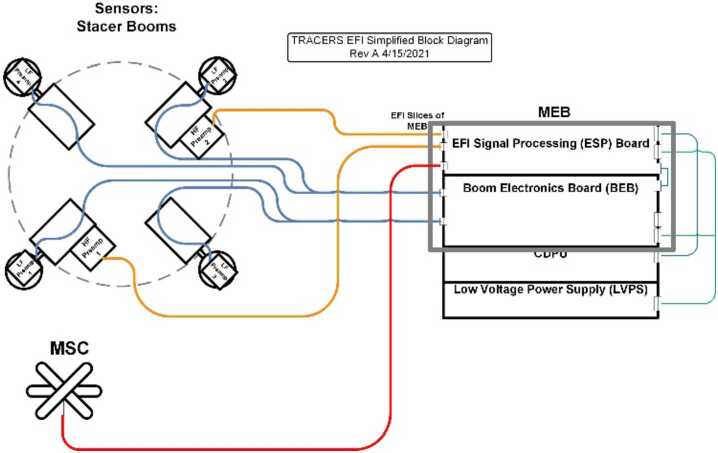
A simplified block diagram of the EFI, showing the sensors, stacer booms, nominal harnessing, and the supporting slices of the TIS MEB, the BEB and ESP. Also shown is the connection to the MSC sensor and preamp assembly

Figures 1 and 2 present schematically the physical implementation of the EFI and its functional elements. As shown in Fig. 1 the four EFI booms are deployed perpendicular to Z-axis of the TRACERS satellite coordinate system (TSCS), in the nominal spin plane of the TRACERS SC, and are nominally aligned with the ± X and ± Y TSCS axes. The signal and power paths running from each of the EFI sensors and booms to the elements of EFI contained in the TRACERS Instrument Suite Main Electronics Box (TIS-MEB), the Boom Electronics Board (BEB) and EFI Signal Processing (ESP) board are shown in Fig. 2. The power and signal path from the Magnetic Searchcoil (MSC) sensor is shown as well. Each of these elements of the design will be described in more detail below, beginning with the sensor and boom system.

### Mechanical Design

The mechanical design of the TRACERS-EFI sensors and deployment systems draws on over 60 years of flight heritage, stretching back to some of the earliest magnetospheric electric field measurements (ISEE), through various auroral, magnetospheric, heliospheric, and planetary missions (CRRES, FAST, Polar, Cluster-II, RBSP, MAVEN, PSP), as well as geospace sounding rockets (GREECE, TRICE-2, INCAA, ACES-2) (Heppner et al. 1978; Mozer et al. 1978; Pedersen et al. 1998; Ergun et al. 2001; Harvey et al. 1993; Gustafsson et al. 1997; Wygant et al. 2013; Andersson et al. 2015; Bale et al. 2016). Figure 1 shows the TRACERS-EFI in fully-deployed configuration. As can be seen in the figure, a rigid stacer boom system is used to deploy the four spin plane sensors. The details of this stacer boom mechanism and the sensors it deploys are described in detail below.

#### Stacer Booms and Sensors

**Fig. 3:**
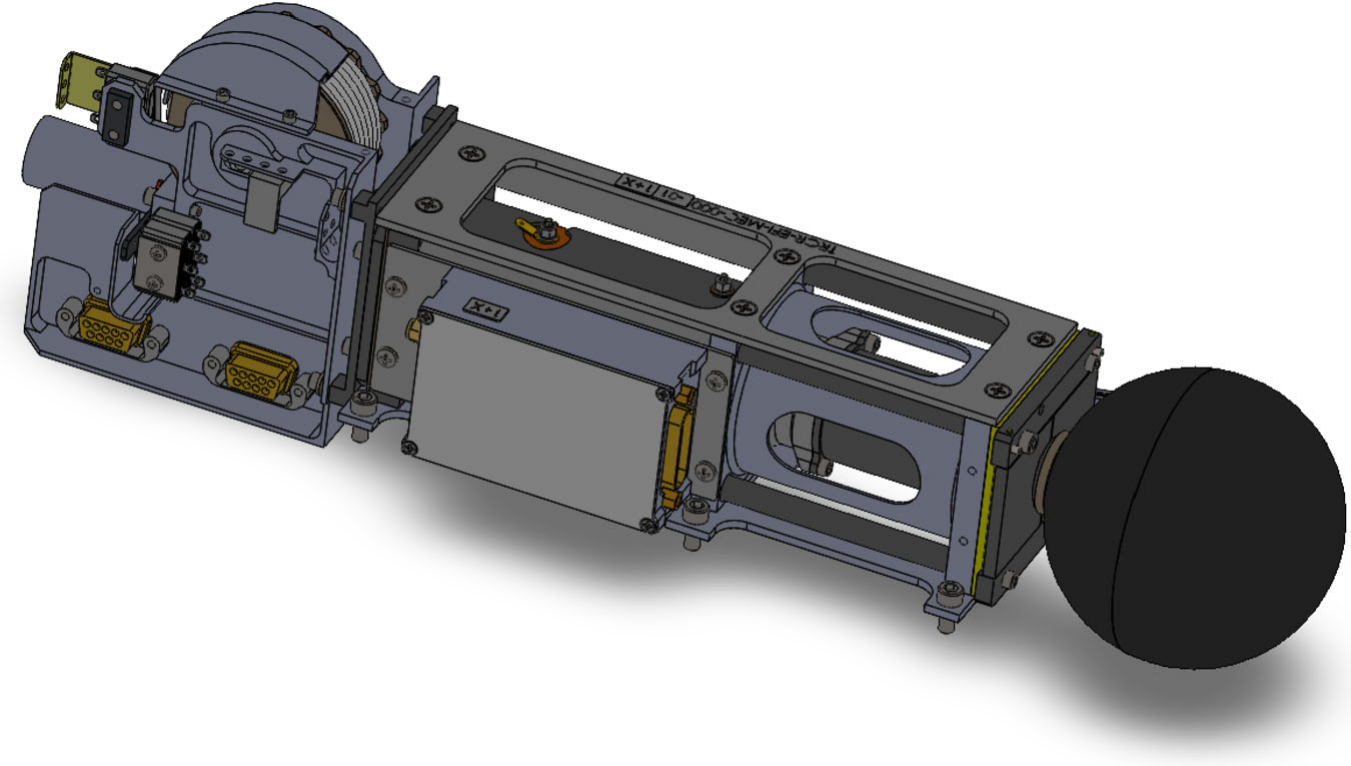
A diagram showing the stowed configuration of one of the four EFI stacer boom units

**Fig. 4:**
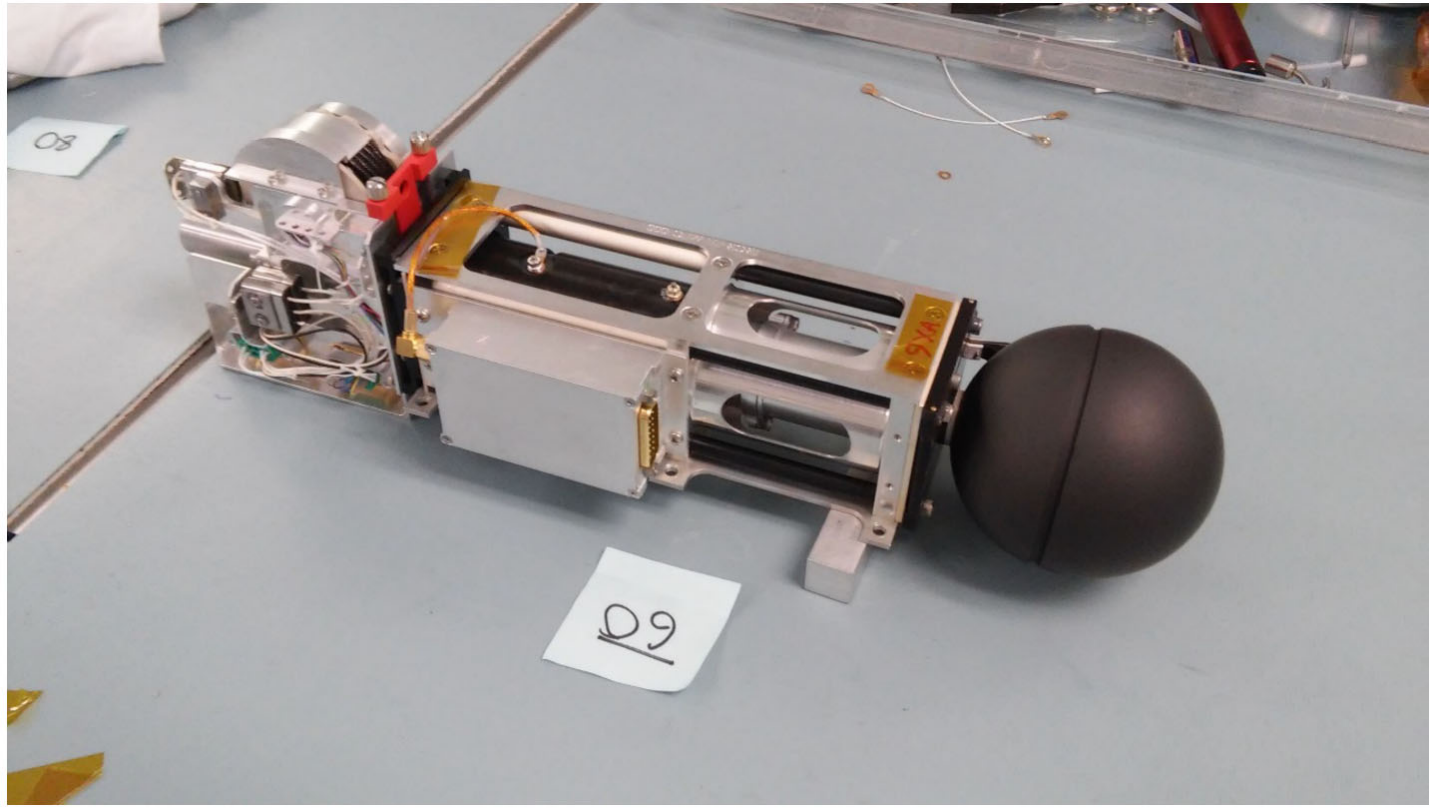
A picture showing one of the flight EFI stacer boom units (SN-09)

A diagram of one of the four TRACERS-EFI stacer booms is shown in Fig. 3, and one of the flight stacer booms is shown in Fig. 4. Each boom and sensor system consists of an 8-cm diameter spherical sensor assembly affixed to the end of a 3.0-m-long DAG-213-coated Beryllium-Copper stacer boom assembly, as well as the stacer deploy assist device (DAD). Each unit has a flight mass of 1.3 kg (2.9 lbs), with the high-frequency preamp (HFPA) assembly adding 0.1 kg (0.2 lbs) to each of the X-axis units. When fully stowed as shown in Figs. 3 and 4, each boom fits in a 37.5-cm by 9.2-cm by 10.6-cm envelope (14.8 × 3.6 × 4.2 inches), including the protrusion of the HFPA enclosure on the ±X-axis units. As described below, when deployed, the spheres at the tips of the stacers and the stacers themselves are electrically isolated from each other and from the DAD ($\ge 1~\mathrm{G}\Omega $), with opposite sensors separated by 7 meters. All four of the spheres are sensors for the low-frequency electric field measurements, and two of the four deployed stacers are sensors for the HF electric field measurements ($\mathrm{EFI}_{1}$ and $\mathrm{EFI}_{2}$, two 3-m long cylindrical sensors with a center-to-center separation of 4 meters. The remaining two stacer booms are connected to chassis ground.

Each spherical sensor consists of an 8-cm diameter graphite-coated (DAG-213) Aluminum sphere with a low-frequency preamplifier (LFPA) mounted inside. As noted above, both the EFI stacer booms and sensor spheres are DAG-213-coated, based on the long history of success with such coatings in magnetospheric and sounding rocket double probe electric field designs. In the past, a single coat of DAG-213 was applied to the Beryllium-Copper stacers and Aluminum spheres in order to reduce the running temperature of those surfaces in sunlight, as well as to provide as uniform a conductive and photoemissive surface on the sensors as possible. However the significantly higher-Atomic Oxygen (AO) densities in Low-Earth orbit relative to those found at higher altitudes in the magnetosphere, combined with the >7-km/s orbital velocity (> 4 eV Oxygen kinetic energy) in Low-Earth orbit (LEO), lead to much higher reaction and erosion rates of coatings like DAG-213 on exposed surfaces than experienced by instruments on magnetospheric missions. To compensate for those losses due to erosion by AO, we increased the number of coats of DAG-213 applied to the stacers and spheres as described below.

To set the required thickness of the DAG-213 coatings on both the spherical sensors and the stacer booms we turned to the experimental results from STS-8 (Visentine et al. 1985), which found the erosion of 4.1 μm of EDAG-106 (equivalent to DAG-213) for a fluence of $3.5\cdot 10^{20}~\mathrm{AO}/\mathrm{cm}^{2}$ On TRACERS, the predicted AO fluence over the original 20-month TRACERS mission at an altitude of 500 km was not-to-exceed $6.5\cdot 10^{20}$ AO/cm^2^ for ram-facing surfaces, and not to exceed $2.1\cdot 10^{20}$ AO/cm^2^ for non-ram-facing surfaces, leading to a worst-case (ram-facing) erosion of 7.5 μm. In the absence of data or evidence to the contrary, we expect the erosion rate to depend linearly on the AO density, and to be distributed uniformly across the surfaces of the spheres and stacers due to the spin of the space craft and the change in AO ram direction over the entire orbit. For the stacers, rather than the usual single coat of DAG-213, three (3) coats were applied for a thickness between 15 and 16 μm (≈ 1-mil) based on SEM imaging of the coated surface. Such a thickness should provide for at least 2 mission lifetimes of wear on orbit. Similarly, each sphere received 10 coats of DAG-213 (50 to 53 μm, or on the order of 3-mil thickness), comparable to that flown on the C/NOFS VEFI instrument in a similar altitude range in LEO (Pfaff et al. 2021), and on the order of 6.6 mission lifetimes.

In order to monitor and account for the changes in the DAG-213 coatings over the mission, we plan to track the electrical effects of erosion using our periodic bias current sweeps to measure the I-V curves of each of the EFI sensors, and using our orbit-by-orbit in flight calibration against $\mathbf{-V\times B}$. Each technique will provide insight into whether the combined effects of the plasma sheath and sensor coatings and their change over the mission has a significant effect on attenuation and effective antenna lengths. That will allow us to correct for those changes over the mission, and even provide a useful case study applicable to future missions in LEO like TRACERS. See Sects. 4.1 and 4.2 below for details of each technique and expected results.

However, we do not expect the incremental changes in the photoemission or resistive properties of the coatings to have a significant impact on EFI performance until many years into the mission. Photoelectrons are not the dominant current carriers in the plasma sheaths under the expected range of densities ($\mathrm{a few times}10^{3}$ to around $5\cdot 10^{5}$ $\mathrm{1/cm^{3}}$) and temperatures (0.1 to 0.5 eV) and so while we will be able to monitor changes in that emission through routine bias current sweeps, and so change in should not have a first-order impact on sensor floating potential and biasing. The surface and through resistance of even a single coat of the DAG-213 coating (hundreds of ohms to a few k${\Omega}$ is several orders of magnitude smaller than the expected sheath resistances (≤100 k$\Omega $ to a few tens of M$\Omega $, and the effective DC input impedance of the LFPA ($\approx \mathrm{G}\Omega $, and so should not represent a significant change in the DC gain of the system.

In the time since the stacer and sensor coating specifications were determined (Nov 2022) the TRACERS orbital altitude was increased from 500 km to 590 km, and the nominal mission duration shortened from 20 to 12 months. The falloff of atomic oxygen density with altitude (≈ a factor of 2 based on NRLMSIS2000 modeling (NRL 2021)), and increased orbital period (fewer orbits per month) increase these lifetime estimates by a factor of ≈2.2.

A custom multi-conductor cable provides the electrical connections between the LFPA and the EFI Boom Electronics Board (BEB) in the TIS-MEB. As in previous sounding rocket and spacecraft designs (GREECE, TRICE-2, MAVEN) when stowed, this cable is stored tightly and compactly on the cable spool visible in the upper left portion of Fig. 3. As in two prior satellite missions (the NASA THEMIS Electric Field Instrument (THEMIS-EFI) and NASA Van Allen Probes (RBSP) Electric Field and Waves Instrument (RBSP-EFW)) a multichannel slip ring provides the electrical connection across the axle of the cable spool. A flyweight brake mechanism integral to the spool limits the deploy rate of a given stacer to between 58 and 62 cm/s (50-100 cm/s allowed range). This deploy rate reduces the Coriolis bending force on the deploying stacer and the end of travel jerk experienced by the cable.

This multi-conductor cable utilizes the same custom Gore design as that flown on the NASA THEMIS-EFI instrument’s spin plane booms (Bonnell et al. 2008), and consists of the following elements, with the addition of a final insulating jacket added to the existing cable stock: An inner coax (AWG-36 single conductor with Al-Mylar over wrap shield layer, 100-pF/m), surrounded by eight insulated single conductors (AWG-36), a Kevlar load-bearing braid, an outer braid consisting of a continuous, helically-wrapped aluminized Mylar shield surrounded by a silver-plated copper braid, and a final insulating jacket, and is approximately 2.5 mm in diameter (0.100 inch).

Each deployed stacer has an exposed length of three (3) meters and a root diameter of 2.5 cm and tip diameter of 1.5 cm. As noted above two of the four deployed stacers (+X and −X) serve as antenna elements for the high-frequency snapshot receiver. Each of these two stacers is connected to its own high-frequency preamplifier (HFPA) mounted on the chassis of the stacers deployer. Standard coax and shielded twisted-pair electrical harnessing connects the HFPAs to the EFI-ESP board on the TIS-MEB. The HFPA and its electrical connections to the stacer boom itself can be seen in the middle foreground of Fig. 4, which shows the stowed configuration of one of the flight EFI stacer boom units (SN-09).

**Fig. 5:**
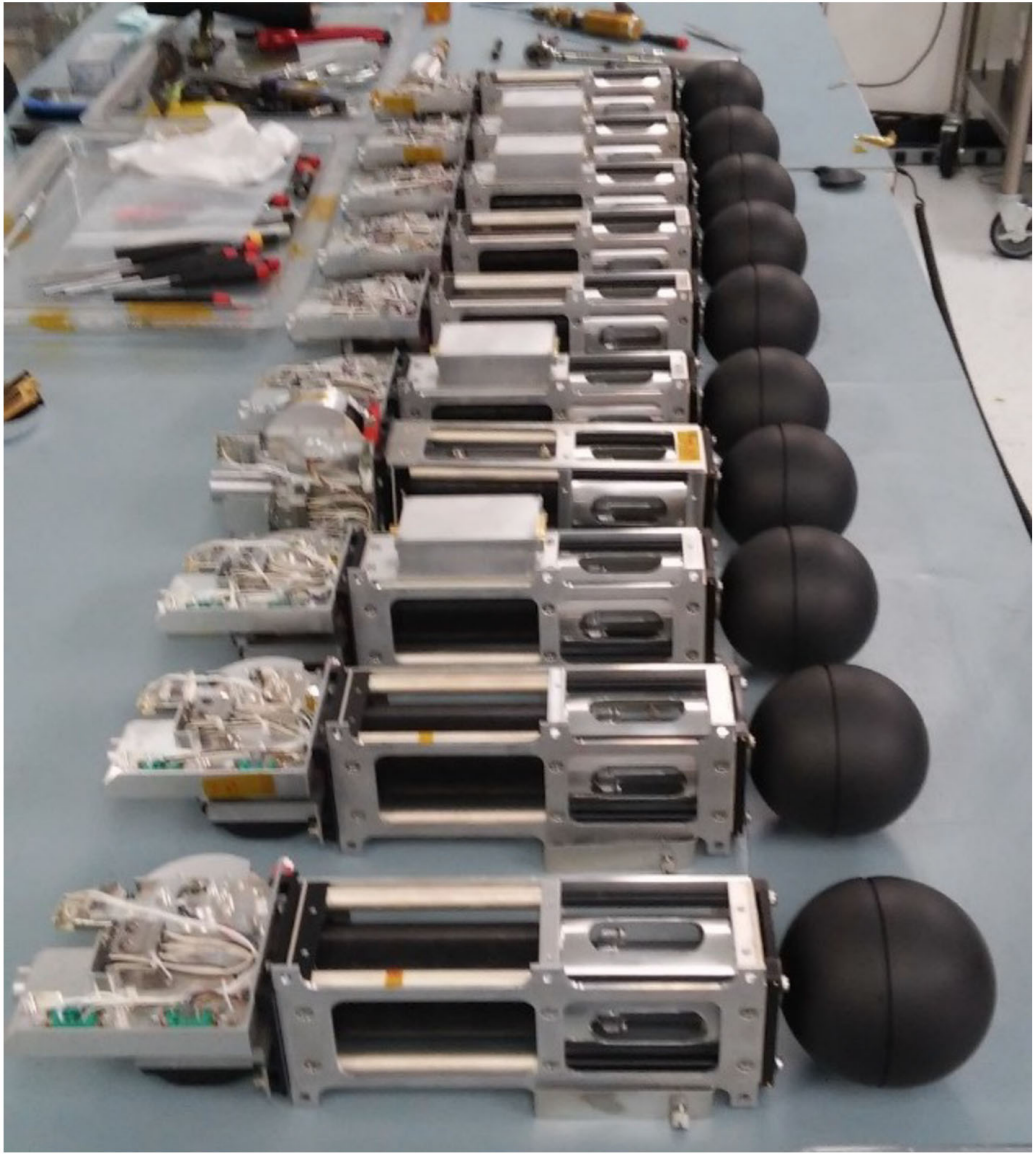
A picture showing all of the flight (8) and flight spare (2) EFI stacer boom units

Figure 5 shows an ensemble shot of all eight (8) flight and two (2) flight spare EFI stacer boom units produced for the TRACERS mission. Aside from the addition of the HFPA and its electrical connections to the stacer boom on four (4) of the flight units, all 10 of the flight and flight spare units are of identical design and function.

### Electrical Design

**Fig. 6:**
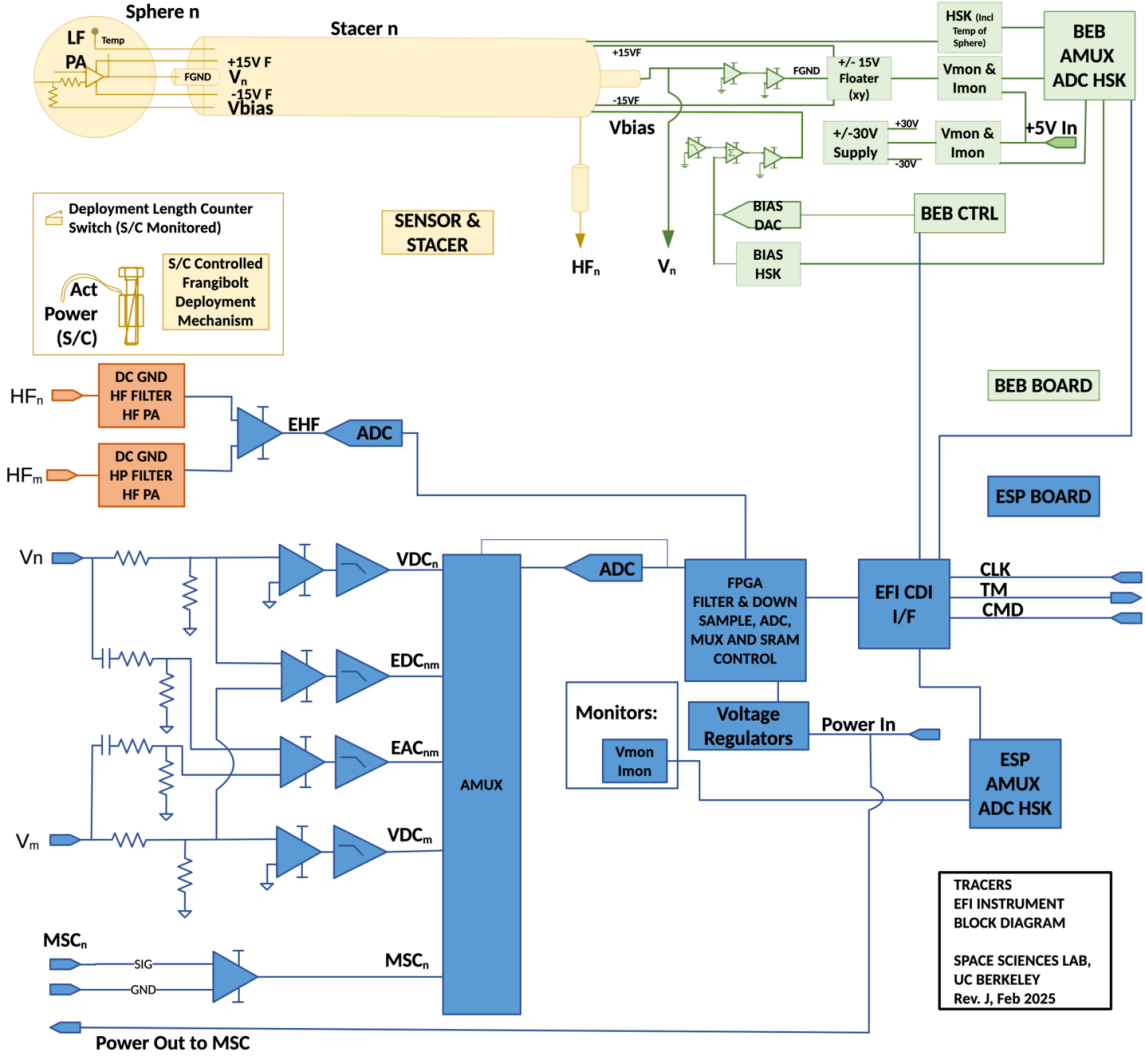
A simplified functional block diagram of the EFI, showing the electrical connections between and functions provided by the sensors, low-frequency preamps (LFPAs), high-frequency preamps (HFPAs), Boom Electronics Board (BEB), and E-field Signal Processing Board (ESP). Also shown are the electrical connections between the ESP and the MSC sensors and preamps

**Fig. 7:**
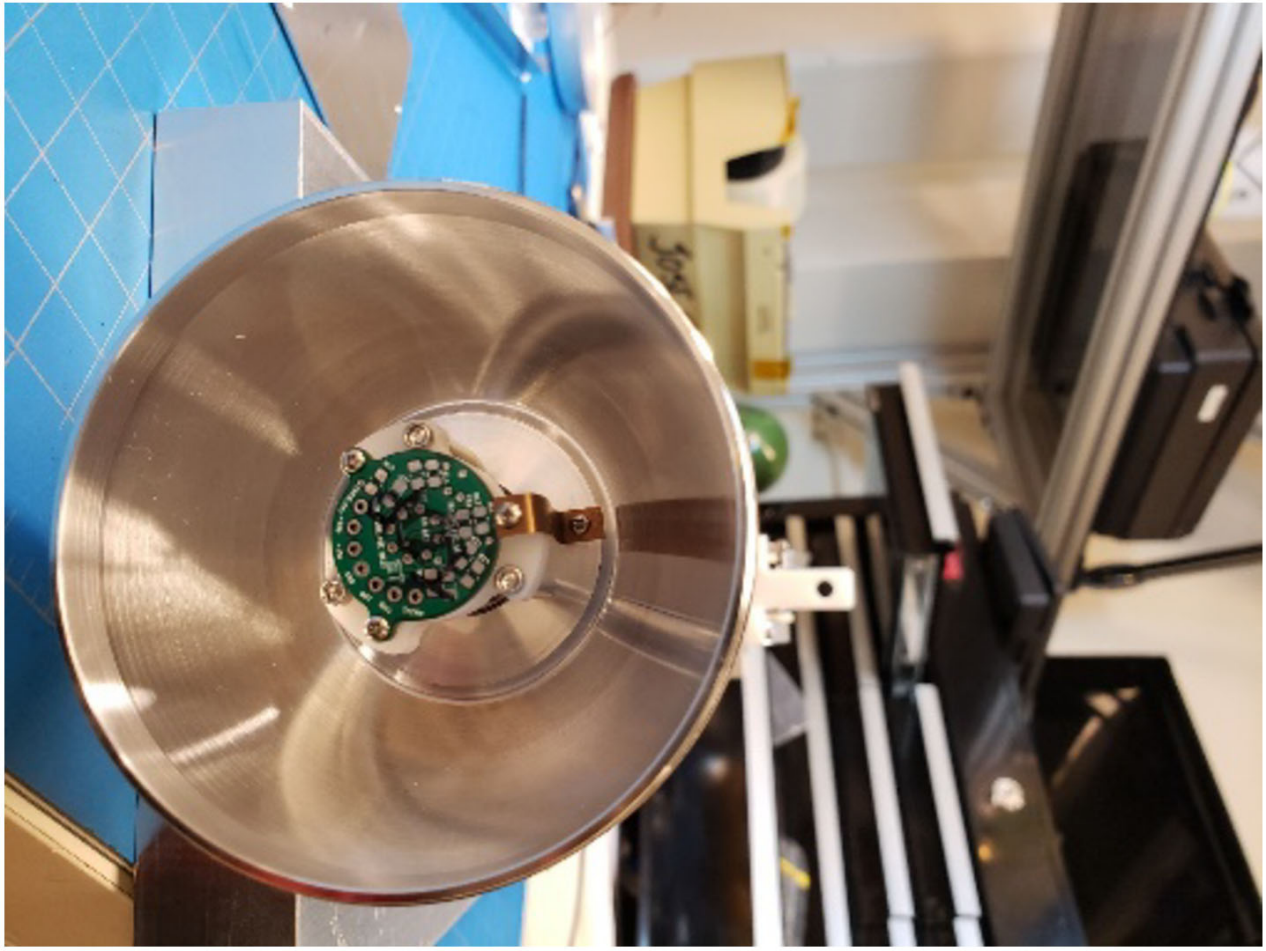
A picture showing one of the LFPAs mounted inside the inboard half of its spherical sensor surface

The electrical design of the EFI is as important as the mechanical design presented above. An electrical block diagram of the TRACERS-EFI is shown in Fig. 6. The EFI follows a basic instrumentation amplifier design for each of the axes, and for both the low- and high-frequency (LF and HF) signal paths. For the LF signal path, each sensor electrode is connected to the input of high-input-impedance ($10^{12}~\Omega \parallel 6~pF$), low-noise unity-gain preamplifier circuit hosted on the LFPA mounted inside of each sphere. Fig. 7 shows how the LFPA is mounted inside of the inner hemisphere of the EFI sensor.

The LFPA circuit drives the buffered sensor potential down the cable to the spacecraft into the BEB and ESP boards of the TIS-MEB, and also serves as the point where the sensor bias current is injected. Each preamp operates off of a separate ±15-V floating power supply, the floating ground of which is derived from a low-pass-filtered version of the preamp output itself. This allows for the use of low-noise, low-input-bias-current, rad-hard (100-kRad(Si)) op amps in the preamp circuit (OP-15, in this case, with input bias currents in the few pA range), while still allowing for the large dynamic range of sensor to spacecraft potentials that can occur due to varying illumination and plasma conditions and current biasing of the EFI sensors.

Each of the two HFPAs connects directly to one of the EFI stacer booms via coax at the root of the boom in the deployer. Each HFPA’s input is AC-coupled to its stacer and powered from a $\pm 5~\mathrm{V}$ power supply as shown in Fig. 9.

#### Sensors and Preamps

**Fig. 8:**
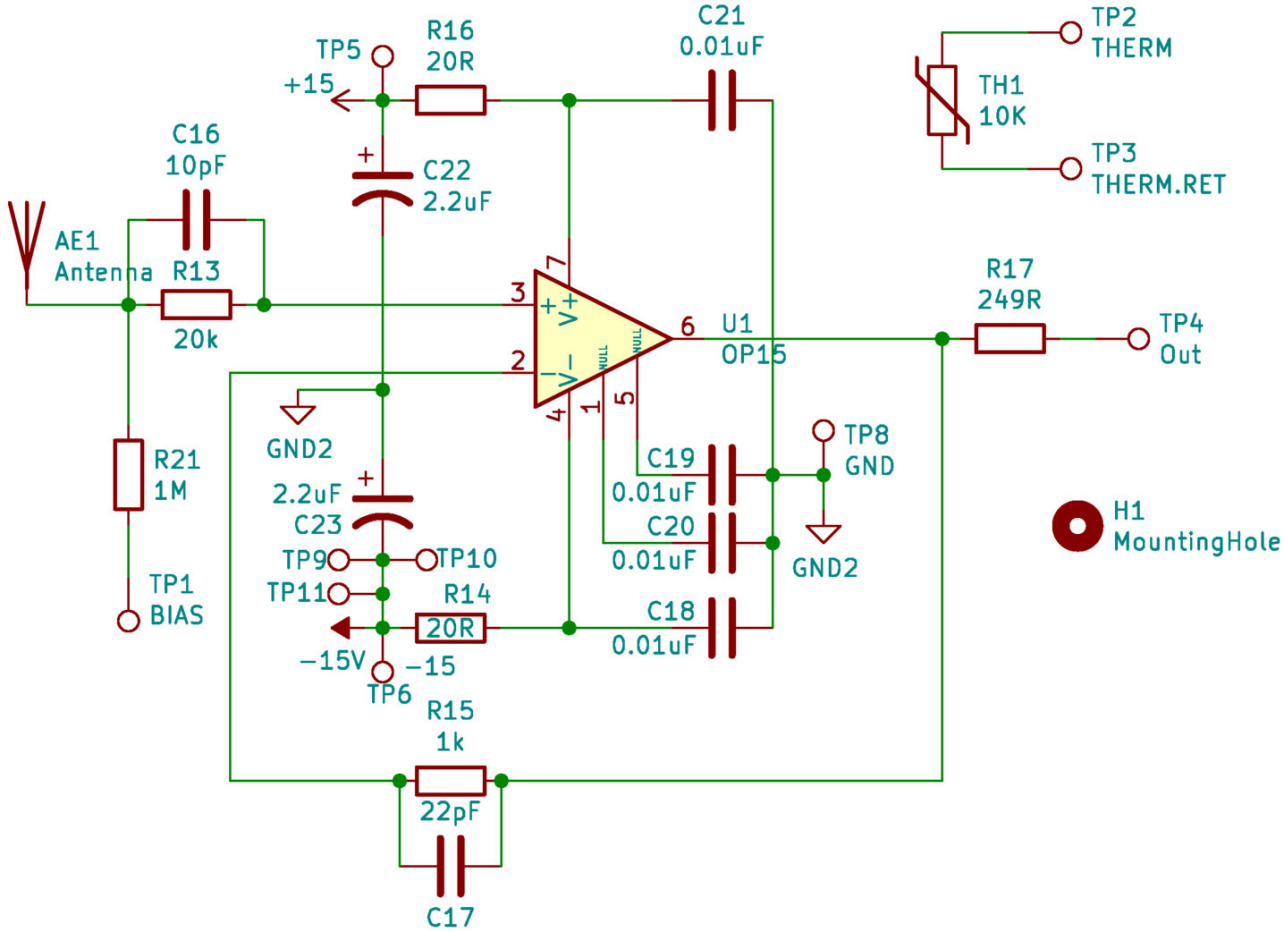
A schematic diagram of one of the EFI LFPA circuits

The EFI LFPA design follows the design flown on several prior magnetospheric and heliospheric missions (THEMIS-EFI, RBSP-EFW, PSP-Fields) (Bonnell et al. 2008; Wygant et al. 2013; Bale et al. 2016). As shown in Fig. 8, it consists of a single op amp (OP-15) in a unity-gain follower configuration, powered via a floating-ground ±15-V supply, with a low-pass roll off frequency of ≈500 Hz. A 20-k$\Omega $ resistor in parallel with a 10-pF capacitor provides for input ESD protection and response compensation, while the 249-$\Omega $ output resistor mitigates the attenuation and stability issues involved with driving the modest ≈0.3 nF capacitive load due to the stacer cable coax. The sensor current bias injection path is via the 1-M$\Omega $ resistor tied to the sensor node and to the VBIAS output corresponding to that sensor from the BEB. The details of that current bias driver circuit will be discussed in the BEB section below.

Temperature excursions of the preamp and sphere — down to −60 deg C in eclipse, and up to +60 deg C in full sunlight — were predicted to occur during each TRACERS orbit throughout the mission due to TRACERS’s nearly noon-midnight-aligned orbital plane. These temperature excursions, while wider than that experienced by the other elements of the EFI, are still well within the −160 deg. C to +90 deg. C range used for qualification and acceptance testing of the LFPA on prior missions. Because the qualification and acceptance testing ranges for the LFPA were broader than those of any of the other EFI hardware, the Flight Model (FM) and Flight Spare LFPAs and spheres were thermal cycled in vacuum (TVAC testing) prior to and separately from the rest of the EFI stacer boom hardware. In this testing, all the flight and flight spare LFPA were subjected to eight (8) cycles from −75 to 75 deg C.

As part of the heritage LFPA design, a fully-enclosing 1-mm-thick Tantalum radiation shield around the OP-15 provides for a total shielding mass equivalent to ≈6 mm of Al, reducing the expected total dose on the OP-15 to less than 1 kRad(Si) (SPENVIS modeling with a radiation design margin of 1) for the nominal TRACERS mission. Originally, this design feature was driven by a requirement to maintain the low nominal input bias current over the entire THEMIS and RBSP mission durations at much higher dose rates and total doses than expected on TRACERS; while the OP-15 is qualified rad hard (to 100-kRad(Si) total dose), increases in DC input bias current and the fluctuations thereof begin to appear at the 40-50 kRad(Si) total dose level (Sahu and Kniffen 1998). While not strictly necessary for TRACERS, this radiation shield is part of the standard design, and provides for a huge lifetime margin for this critical component of the LF signal path.

**Fig. 9:**
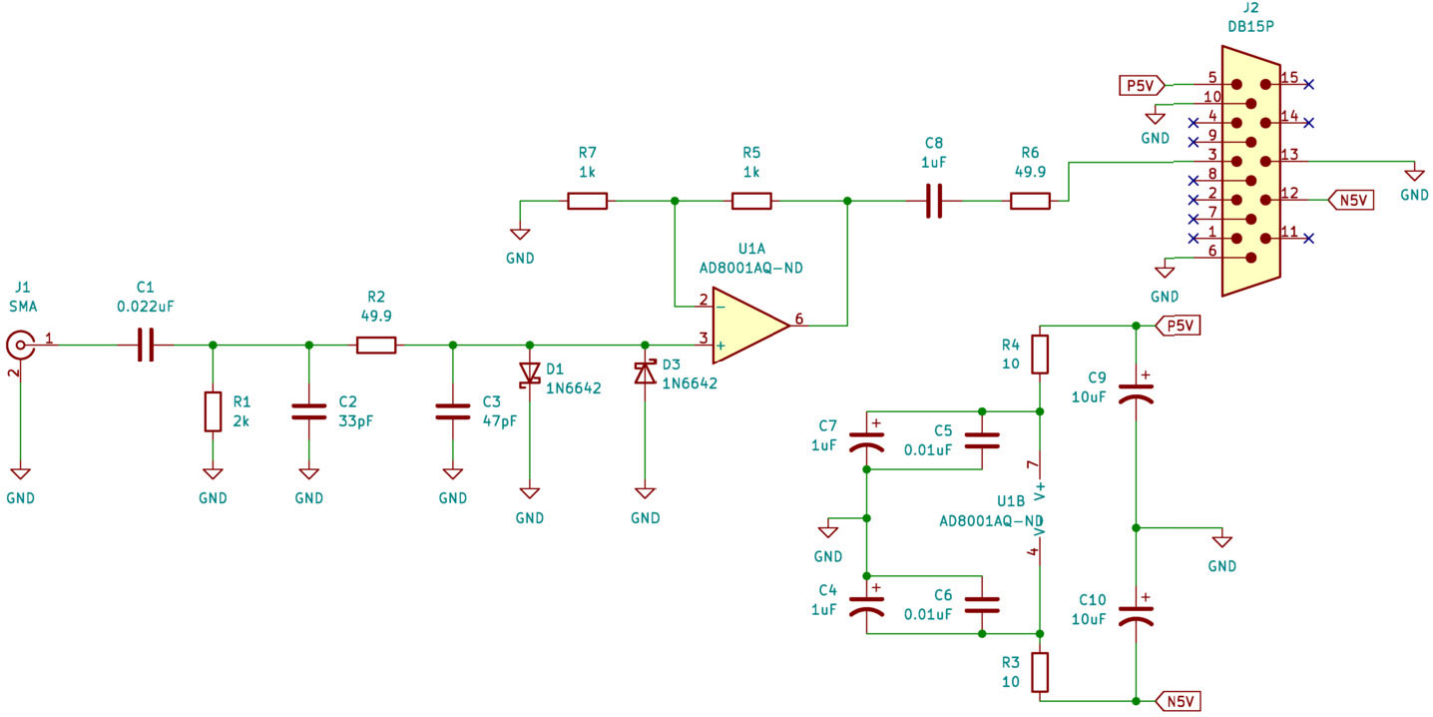
A schematic diagram of one of the HFPA circuits

**Fig. 10:**
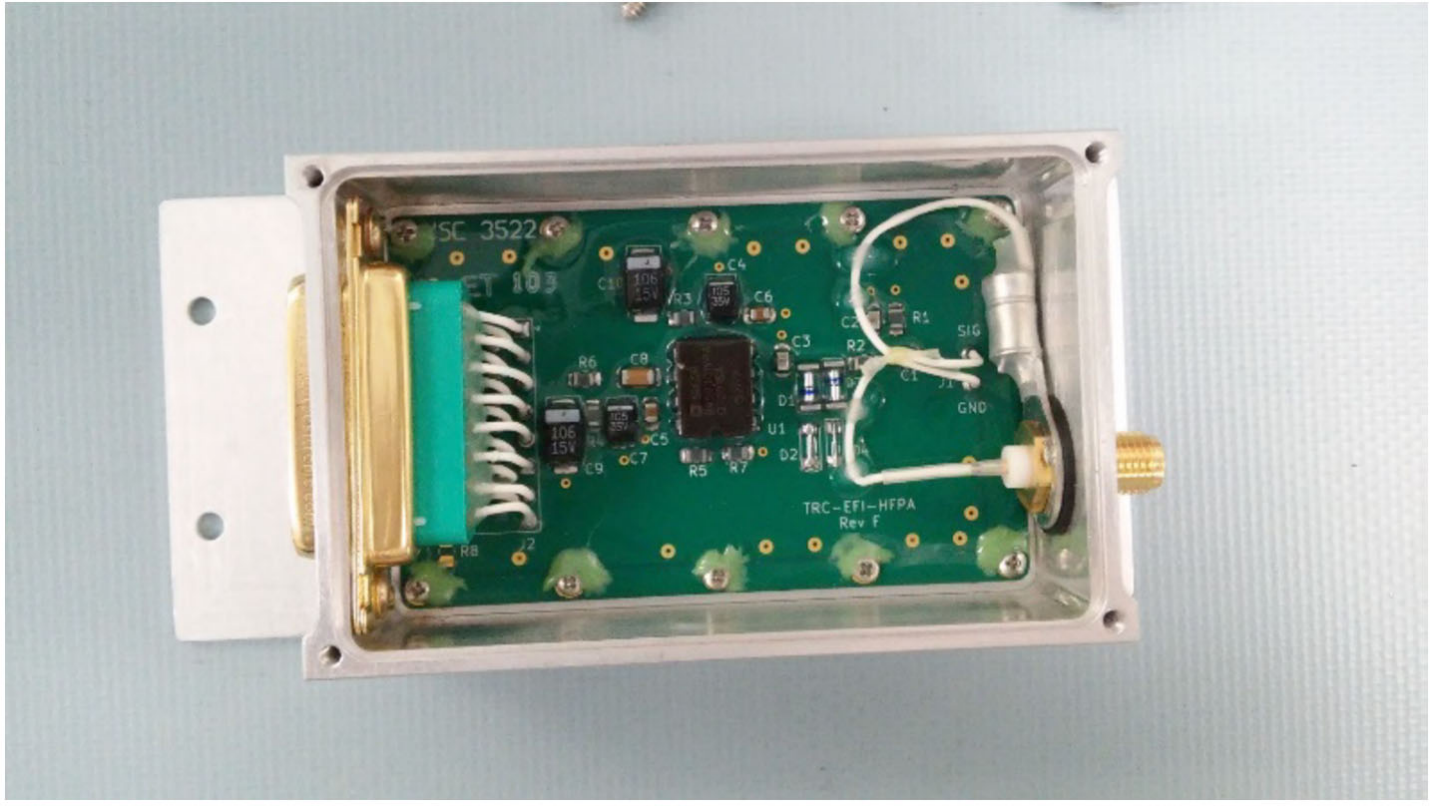
A picture showing a HFPA with its cover removed prior to installation to the stacer boom deploy unit

The EFI HFPA design follows that flown on the PSP Fields mission (Bale et al. 2016) with some simplifications allowed by the separate LF and HF antenna elements on TRACERS-EFI. Figure 9 shows a schematic of the HFPA, and Fig. 10 shows the top side on one of the HFPA circuit boards inside its enclosure. A passive RC input network to the HF op-amp (AD8001) blocks the lower-frequency variations in the HF sensor (stacer boom) potential that arise due to spin-dependent illumination and plasma flow aspect angle. A diode clamp keeps the input potential variations within a nominal ± 0.7 volt range (±1.4 V maximum at high current or anomalously low source impedance) to protect the op-amp input.

**Fig. 11:**
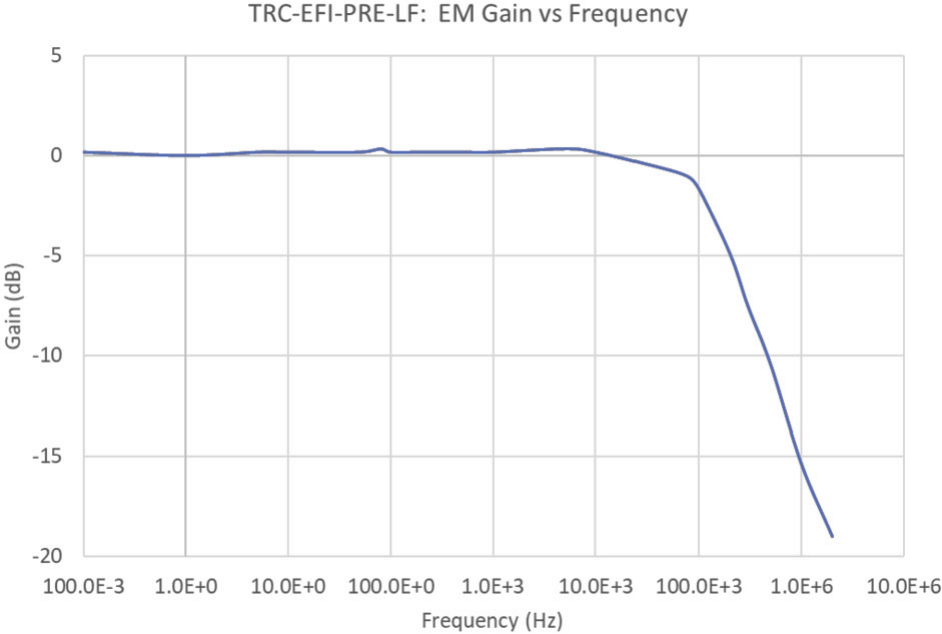
Frequency response of the LFPA from 0.1 Hz to 1 MHz

**Fig. 12:**
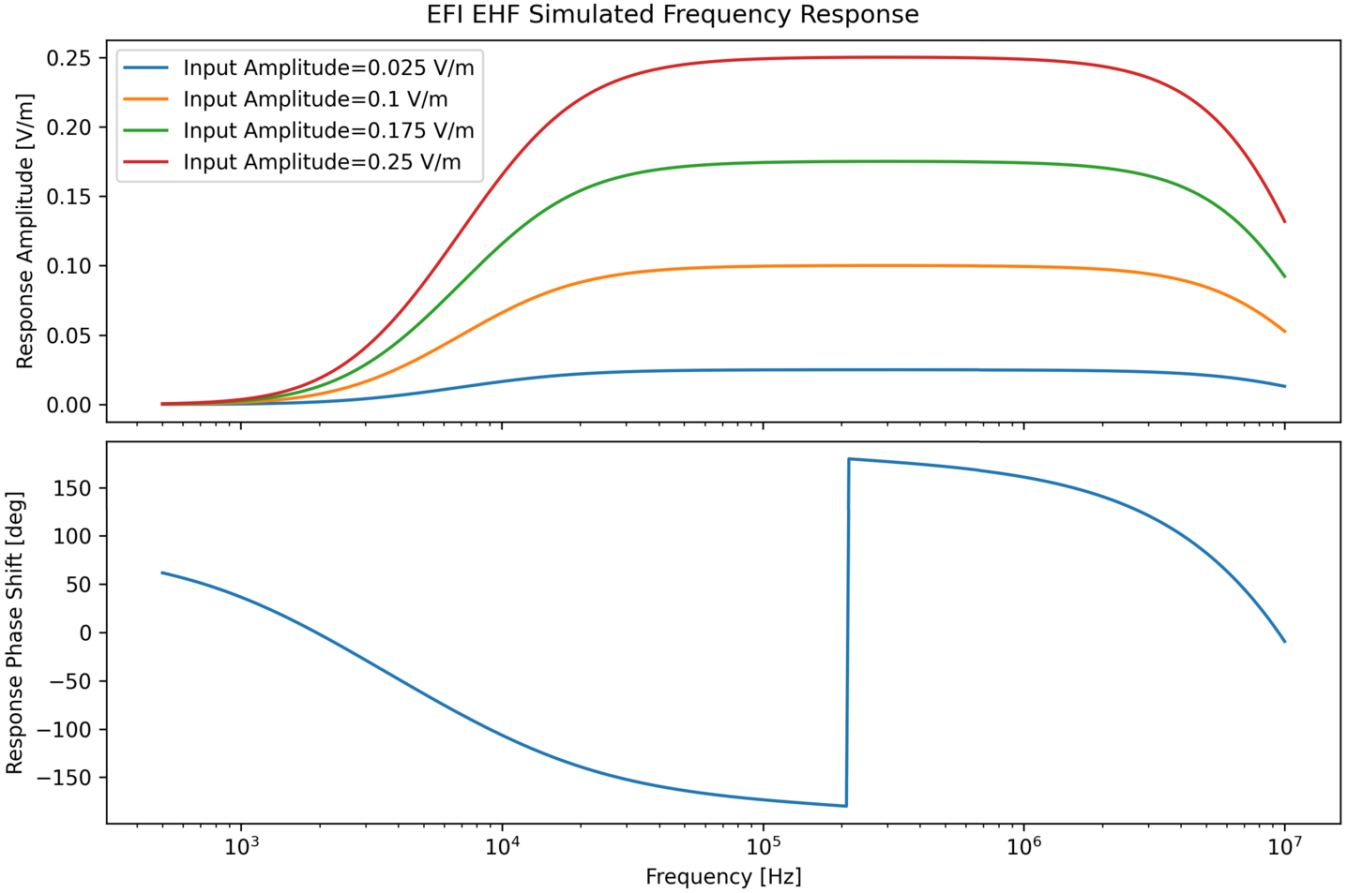
Frequency Response of the HFPA and EHF signal processing on the ESP board from 500 Hz to 10 MHz. Top panel shows the output amplitude for a selected set of input amplitudes and shows the voltage gain of 1 in the passband between 10 kHz and 10 MHz. The bottom panel shows the phase shift; note that the actual phase shift is a smooth function of frequency, and the abrupt swing of the phase from −180 deg to 180 deg near 200 kHz is an artifact of the arctan function used to compute the phase from the complex voltage gain

The expected frequency response of the LFPA in the ambient plasma and illumination conditions in the TRACERS science orbits is shown in Fig. 11. The LFPA’s high input impedance combined with the relative low sheath impedance leads to a flat frequency response with a voltage gain of 1. the total gain (top panel) and phase shift (bottom panel) as a function of frequency for the HFPA is shown in Fig. 12. The four curves in the top panel show the output amplitude as a function of frequency for four selected input amplitudes, with the broad plateau in each curve showing the voltage gain of 1 within the passband of the circuit, rolling in at around 10 kHz, and rolling off at 10 MHz. The curve in the bottom panel shows the phase shift. Note that the actual phase shift is a smooth function of frequency, and the abrupt swing of the phase from −180 deg to 180 deg near 200 kHz is an artifact of the arctan function used to compute the phase from the complex voltage gain.

#### Boom Electronics Board (BEB)

**Fig. 13:**
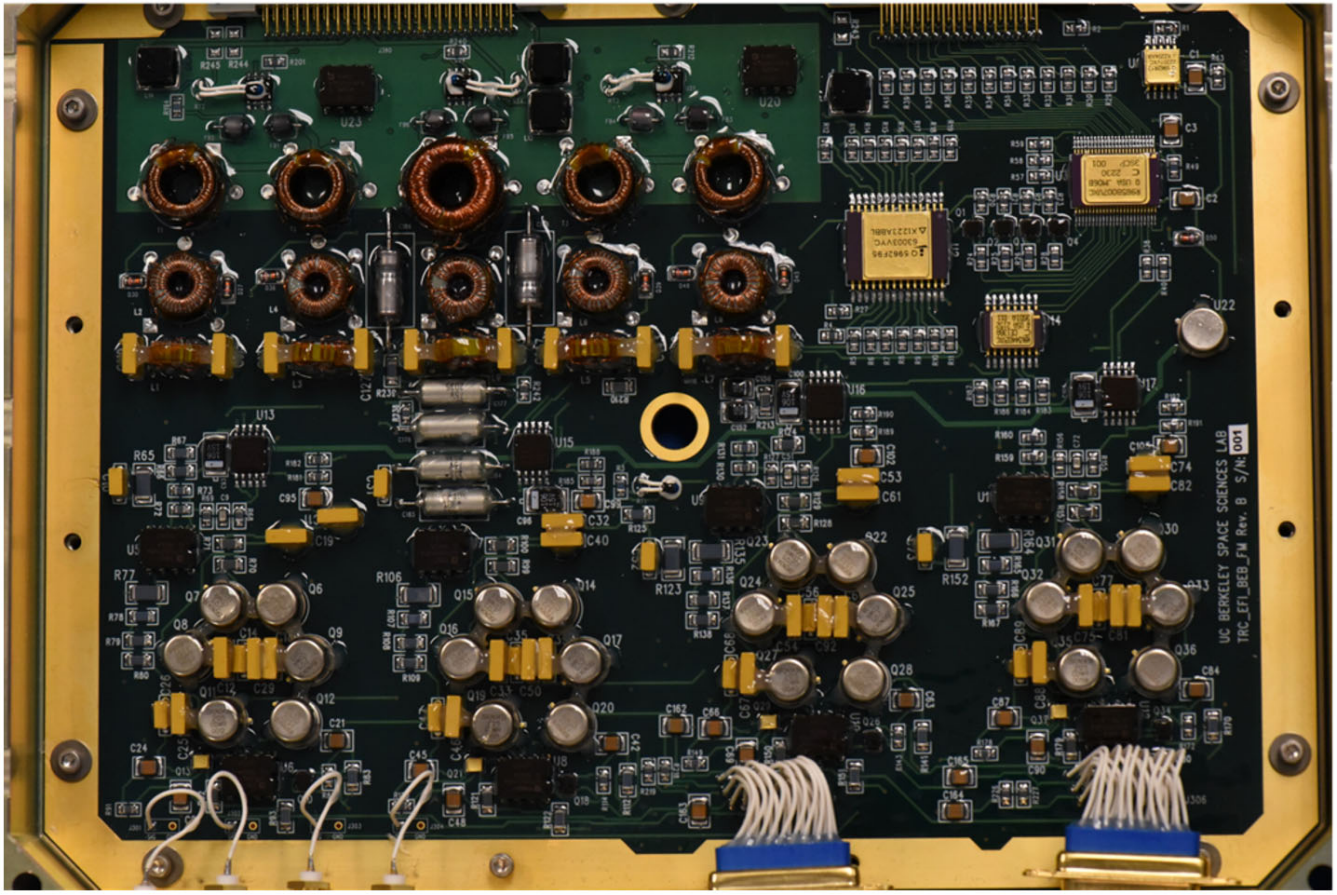
A picture of the top side of one of the flight EFI BEBs

The TRACERS-EFI utilizes a single, centralized Boom Electronics Board (BEB), mounted in the TRACERS Instrument Suite Main Electronics Box (TIS-MEB), with power services provided by the MEB-Low Voltage Power Supply (LVPS). This design choice follows that used on prior missions (THEMIS, RBSP, MAVEN, PSP), and was originally driven by the tight mass and volume budgets for THEMIS, and has since become a standard system architecture for most, if not all, double-probe E-field instrument designs flown on satellites or sounding rockets. Fig. 13 shows a top side view of one of the Flight BEBs.

As is shown in Fig. 6, the BEB is responsible for generating and controlling the floating grounds (FGND), and the current bias signals (VBIAS) for each of the four EFI sensors. Current biasing is controlled by 12-bit digital-to-analog converters (DAC121S101) with a dynamic range of ±22 volts relative to each sensor’s potential up to a maximum of ±22 V (VBIAS_*n*_) relative to SC ground. This allows for the injection of up to ±22 uA/sensor of bias current with a resolution of 11 nA/ADC step. The FGND and VBIAS signals are all referenced to a low-pass-filtered (passive one-pole RC at 500-Hz) version of the sensor potential to allow for stable DC biasing of the various surfaces in the presence of the low-frequency, several-volt excursions of sensor-to-spacecraft potential that occur due to changes in sensor illumination and ambient plasma conditions.

The power and switching used to fire the shape-memory-alloy (SMA) Frangibolt actuators that restrain the stacer booms prior to deployment on orbit, and the click counter read backs from the stacer boom deployments are handled through harnessing connected to the SC avionics box. Each actuator line is carried by a twisted pair in the individual branches of the EFI actuator harness, with the entire harness bundle’s shield terminated to actuator power ground at the Spacecraft Avionics Box.

The BEB also provides the resources for a self-test capability when the EFI sensors are in their stowed configuration. When stowed, the individual sensors are connected to analog ground via spring-loaded conductive polymer (SC-Consil) sensor contacts to local chassis ground (≤1 k$\Omega $). These connections provide for electrostatic discharge (ESD) protection of the LFPA inputs, as well as for a simple DC functional test with a dynamic range of ±15 mV (from −45 to −15 mV) that can be run by commanding the individual sensor BIAS lines through a set of values while collecting waveform telemetry data. This self-test capability was used extensively during TIS and Observatory Integration & Test (I&T), eliminating the need for external electrical ground support equipment (GSE) to support each performance test, and standardizing the instrument configuration for each test run. As shown in Fig. 6, each individual sensor potential is sent in parallel to the BEB circuits, and the analog signal conditioning circuits on the ESP. Connection from the BEB to the ESP is provided by short coax lines and threaded coax panel connectors (SMA-type) on the front panel of the MEB.

#### EFI Signal Processing Board (ESP)

The ESP board provides the command, telemetry, and power interfaces for the EFI. It also provides the sensor and preamp power and analog science signal processing for the MSC instrument described in detail in (Hospodarsky et al. 2025) Fig. 14 shows a top side view of one of the Flight ESP boards. The ESP implements the small set of EFI commands — instrument reset; BEB VBIAS digital-to-analog (DAC) commanding and interrogation; enable/disable of timestamp synchronization to the SC GPS one-pulse-per-second (1PPS) signal; and ESP control and data register interrogation via housekeeping. It also provides for the analog-to-digital conversion of all EFI housekeeping (see list in Table 2 below. The ESP anti-alias filters each of the DC-1 kHz channels (EDC, VDC, EAC) using a four-pole analog Bessel filter. The ESP does not apply any additional analog filtering to the MSC data prior to analog-to-digital conversion. Then, under the control of the ESP’s FPGA, continuous time series data of the VDC, EDC, and EAC analog channels are gathered at 2048 samp/s/channel. Every 488.4-us (≈ 2048 times per second), the 11 input channels are sampled at an inter-channel timing of 5-us (200 ksamp/s, or ≈ 1000 times the sample rate) in the following order: VDC1, VDC2, VDC3, VDC4, EDC12, EDC34, EAC12, EAC34, MSCX, MSCY, MSCZ. The ESP then digitally filters and down samples the EDC and VDC channels in successive factors of two into the final 128 samp/s/channel Region of Interest (ROI) data products. The 8 samp/s/channel Back Orbit (BOR) data products are derived from a 1 in 16 downsampling of the ROI data without filtering. The two EAC channels remain at their full 2048 samp/s/channel time resolution, as do the three MSC channels. The single EHF channel is anti-alias filtered at 10 MHz, and sampled once every 16 seconds at $20\cdot 10^{6}$ samp/s for 16384 samples to generate the HF snapshot data.

**Fig. 14:**
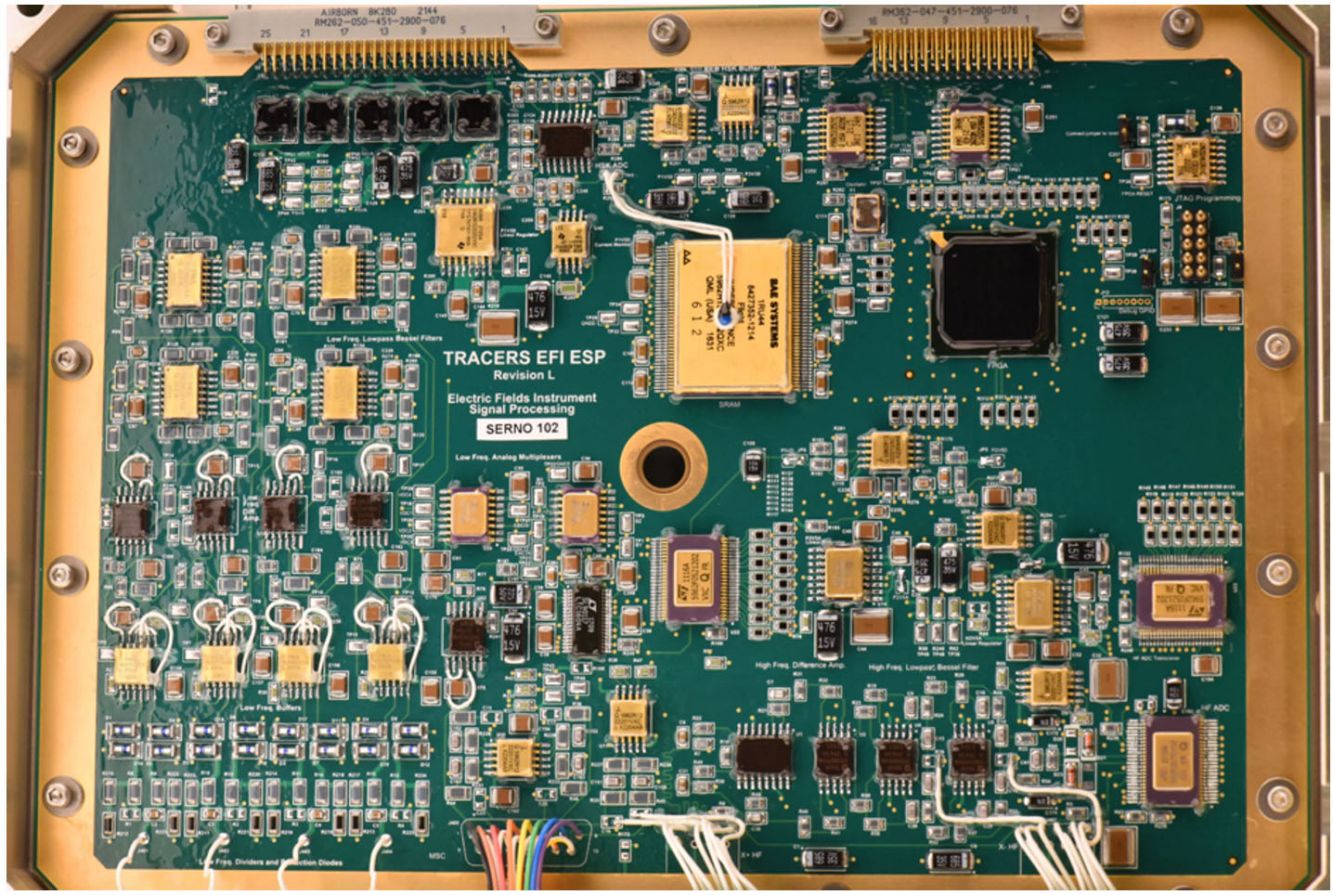
A picture of the top side of one of the flight ESP boards

The ESP then compiles all the time series, snapshot, and housekeeping data into separate data packets (Consultative Committee for Space Data Systems, or CCSDS-format) and transfers them via a serial data interface to the TIS Central Data Processing Unit (TIS CDPU). Depending on the TIS operational mode - Region of Interest (ROI) or Back Orbit (BOR) - the CDPU stores or discards EFI packets by their type to implement the desired data collection strategy, as is described in detail in (Christopher et al. 2025).

### Data Quantities

The data quantities available from the EFI are shown in Table 1.

**Table 1:** TRACERS EFI Data Quantities

Quantity	Dynamic Range	Resolution	ROI Rate	BOR rate
VDC_*n*_, n = 1..6	±45 V	16-bit, 1.4 mV/ADC	128 samp/s	8 samp/s
EDC_12_, EDC_34_	±7.1 V	16-bit, 0.2 mV/ADC	128 samp/s	8 samp/s
	(±1.014 V/m)	(0.029 (mV/m)/ADC)		
EAC_12_, EAC_34_	±4.72 V	16-bit, 0.16 mV/ADC	2048 samp/s	Same as ROI^1^
	(±0.674 V/m)	(0.023 (mV/m)/ADC)		
MSC_*n*_,*n* = *x*,*y*,*z*	±12 V	16-bit, 0.37 mV/ADC	2048 samp/s	Same as ROI^1^
	(±49.6 nT at 100 Hz)	(15 pT/ADC)		
EHF_12_	±1.4 V	12-bit, 0.85 mV/ADC	20⋅10^6^ samp/s^2^	Same as ROI^3^
	(±0.35 V/m)	(0.225 (mV/m)/ADC)		

^1^One (1) 1024 sample long snapshot (0.5-s duration) every 16 s

^2^One (1) 16384 sample long snapshot (≈0.8192 ms duration) every 16 s

^3^One (1) 16384 sample long snapshot (≈0.8192 ms duration) every 2048 s

In addition to the science quantities, the ESP collects a complete set of EFI housekeeping quantities and telemeters them in the EFI housekeeping packet. These housekeeping quantities are summarized in Table 2 below.

**Table 2:** TRACERS EFI Housekeeping Quantities

Quantity	Dynamic Range	Resolution	Comments
IMONs	0 to 500 mA	12-bit, mA/ADC	
VMONs	0 to ±5 V	12-bit, 1.2 mV/ADC	
TMONs	≈±75 deg C	12-bit, variable^1^	
VBIAS	±5 V	12-bit, 24 mV/ADC	
BIASDAC	0 to 4095	12-bit	
ERR FLAGS and COUNTS	n/a	n/a	Various, see ^2^ below.
REGISTER ADDR and VALUE	n/a	n/a	

^1^Mapping of TMON ADC counts to temperature is monotonic but non-linear. For the ESP and LFPA TMONs the resolution varies from 0.033 to 0.2 deg C/ADC (cold to hot). For the BEB TMON the resolution varies from 0.015 to 1 deg C/ADC (cold to hot)

^2^CMD_COUNT, CMD_ERR, PPS_ERR, CMD_FRAME_ERR, CMD_PARITY_ERR, MEM_ERR_CNT, TLM_SRAM_TIMEOUT, BEB_FIFO_OVERFLOW, HF_ADC_OVERFLOW, EDC_B_PKT_ERR, VDC_B_PKT_ERR, EDC_R_PKT_ERR, VDC_R_PKT_ERR, MSC_PKT_ERR, EAC_PKT_ERR, CMD_SYNC_ERR

Once on the ground, the TRACERS and EFI Science Operations Centers (SOC) process the raw EFI telemetry into calibrated science quantities in physical units (e.g. V, V/m, $(\mathrm{V/m})^{2}/\mathrm{Hz}$). The vector E-field quantities EDC and EAC will be available in several operationally or geophysically relevant coordinate systems and frames of references as described in detail in the TRACERS SOC paper (Christopher et al. 2025). In particular, the data in the geophysical coordinate systems (geographic, geomagnetic field-aligned) will be available in both the frame co-moving with the TRACERS spacecraft, as well as that in the Earth-Fixed co-rotational frame where the $-\mathbf{V \times B}$ E-field arising from the motion of the TRACERS SC frame across the Earth-fixed background model magnetic field has been removed.

## On-Orbit Operations

Once the EFI stacer booms are deployed, the only nominal EFI operations will be regular IBIAS sweeps, updates of the dayside and nightside BIAS settings based on the results of those IBIAS sweeps, and routine, continuous on-orbit cross-calibration of the E-field estimates from EFI against those derived from the SC velocity, the local model or measured magnetic field, and the strong (up to 360 mV/m) $-\mathbf{V \times B}$ E-field present in the SC frame of reference. Both of these routine activities are key to measuring the gains, offsets, and other calibration factors that allow EFI to achieve the required accuracy in the ROI. The current biasing, bias sweeps, and on-orbit cross-calibration of EFI data are discussed in detail in the following subsections.

### Sensor Bias Current Sweeps and Bias Optimization

The nature of the plasma sheaths that couple the EFI sensors to external electric fields (gradients in the plasma potential) and plasma density variations are similar to those experienced in the more tenuous and hotter plasmas of the magnetosphere and heliosphere but differ in some important details. Those similarities — the dependence of the sensors floating potential relative to the local plasma potential on the balance of currents flowing to that sensor and how that floating potential depends on sensor illumination, plasma flow aspect angle, and ambient plasma density — drive the requirement to current bias the sensors to optimize EFI’s operating point on the current versus voltage, or I-V, curves of those sheaths in the presence of any illumination or plasma wake effects that arise because of the TRACERS SC themselves.

A key difference between the LEO and magnetosphere/heliosphere cases lies in the relative magnitudes of the currents that arise from the ambient plasma electrons and ions (typically O^+^ and H^+^ at TRACERS altitudes), and those from photoelectron emission. In the terrestrial ionosphere at TRACERS altitudes, the ambient plasma densities are much higher than those in the magnetosphere and heliosphere — a few times $10^{3}$ to a few times $10^{5}$ $1/\mathrm{cm}^{3}$ versus 0.1 to a few $1/\mathrm{cm}^{3}$. This leads to the thermal current of the ambient electrons outweighing that from photoelectron emission or the cold ion flow in the SC frame (typically supersonic for O^+^, transonic for H^+^) by an order of magnitude. In this situation, we expect to have to balance the incident electron current (electrons to the sensor) against the applied bias current (electrons from the sensor), with the source of the bias current being current collection across the exposed current-conducting surface of the SC.

Another key difference between the LEO and magnetospheric/heliospheric cases lies in the higher magnetic field strengths, higher densities, and lower plasma temperatures, resulting in Debye shielding lengths and electron gyroradii on the order of or even less than the sensor and SC dimensions. This should lead to thinner plasma sheaths with less focusing effects as well as significant anisotropies in electron current collection. Unfortunately, there are no simple, accurate, analytical models available for the plasma sheath’s current-voltage relationship, traditionally called I-V curves, at these intermediate ratios of sensor dimensions and plasma scale lengths (Patacchini and Hutchinson 2011). To work around this uncertainty, our approach has been to accommodate what should be the largest ambient currents (un-magnetized thermal electrons in the thick-sheath limit at the peak environmental densities, at around 10 to 20 $\mu $A/sensor) while maintaining the option of applying either polarity of bias current to the sensors with a fine enough resolution to accommodate the factors of 2 to 5 reduction that magnetized current collection effects should cause.

In order to gather the I-V curve data for each sensor required to set the optimal bias currents and to monitor the resistive and photoemissive behavior of each sensor over time, the CDPU commands the EFI to sweep the bias currents on opposing sensor pairs over a selected range of values while keeping the bias currents of the other opposing pair fixed at their nominally commanded values. These sweeps occur twice per day per SC initially (once in sunlight, once in eclipse) in order to determine the initial bias current settings and to track any changes in the current collection and thus floating potentials of the sensors and SC surfaces as they interact with the ambient plasma, neutral atmosphere, and other environmental factors. Once any trends stabilize, probably within the first few weeks on on-orbit operations, the bias sweep cadence will drop to two per week per SC.

### Cross-Calibration and Determination of Offsets and Boom Shorting Factors

Measuring the DC geophysical E field in LEO is challenging - the required accuracy of $\pm 3~\mathrm{mV}/\mathrm{m}$ has to be achieved in the presence of a significant background field of up to $\pm 360~\mathrm{mV}/\mathrm{m}$ in the SC frame, or a relative uncertainty of no more than $\pm 0.9\%$. Many of the calibration parameters that affect the E-field estimate can be measured on the ground to accuracies better than this: The expected lengths and alignments of the EFI sensor booms; the expected resolution and accuracy of the spacecraft attitude; the electrical offsets and gains in the EFI signal processing paths. Other parameters are practically impossible to model or measure accurately enough on the ground – for example, the “shorting factor” of the antennas, stray fields due to wakes or photoelectron clouds around the spacecraft and booms, and transients caused by the shadowing of the sensors by the SC, either in terms of solar illumination or particle fluxes along B. Estimating the on-orbit values of these calibration parameters is key to the accurate estimate of the geophysical E field by EFI in the ROI, and throughout the full orbit as well. In order to accurately estimate and account for these effects, we will use continuous, full-orbit cross-calibration of the EFI DC data against the $\mathbf{-V\times B}$ present in the SC frame due to its motion across the background geophysical B field. The details of this cross-calibration process are described below.

The electric field measured in the TRACERS SC frame by the EFI instrument, $\mathbf{E}'_{\mathrm{EFI}}$, is related to that present in the Earth-fixed coordinate system and reference frame by the relation: $\mathbf{E}'_{\mathrm{EFI}} = \mathbf{R}_{\mathrm{EFI}}\cdot \mathbf{R}_{\mathrm{SC}}\cdot \mathbf{E}' = \mathbf{R}_{\mathrm{EFI}}\cdot \mathbf{R}_{\mathrm{SC}}\cdot (\mathbf{E} - \mathbf{V \times B})$, where $\mathbf{R}_{\mathrm{EFI}}$ represents the rotation matrix from the SC to the EFI coordinate systems (i.e. any rotation or misalignment between the two EFI axes and the SC coordinate system); $\mathbf{R}_{\mathrm{SC}}$ the rotation matrix from the SC coordinate system to the Earth-fixed coordinate system (i.e. the attitude solution for the SC provided on the ground by the SC-side Guidance, Navigation, and Control (GNC) system); $\mathbf{E}$ and $\mathbf{B}$ the E-field and B-field in the Earth-fixed frame (i.e. the geophysical E and B fields); and $\mathbf{V}$ the relative velocity of the SC frame and Earth-fixed frame (i.e. the SC orbital velocity in the Earth-fixed frame).

In addition to the factors that depend on geophysical fields, SC orbit, SC attitude, and instrument alignment angles, there are offset and scaling factors that relate the potential differences measured between the EFI sensors and $\mathbf{E}'_{\mathrm{EFI}}$. These EFI calibration factors, such as the so-called boom shorting factor, offsets arising from spin-dependent differential current collection (cold flowing ions), wake effects, or transients caused by shadowing of an EFI sensor by the SC may be estimated by a careful cross-calibration of measured E field against that predicted from SC orbital motion and the main geomagnetic field.

The boom shorting effect is a well-known, but difficult to accurately predict, electrostatic effect observed on double probe E-field instruments in magnetospheric or heliospheric environments that arises from the redistribution of charge on the spacecraft and EFI boom systems in response to a given external E-field (Pedersen et al. 1998; Bonnell et al. 1997; Wygant et al. 2013; Califf and Cully 2016). Even though the EFI sensors are electrically isolated from the spacecraft, the presence of the conducting stacer booms and spacecraft between the two EFI sensors making up a given axis of measurement reduces the potential difference between those two sensors from that would be present in the absence of the boom system, effectively shorting out a portion of the external field. While Debye shielding effects in the cold dense ionospheric plasma in the TRACERS environment are known to minimize this shorting effect (Pfaff et al. 2021), comparison between the observed magnitude of E measured by EFI ($\mathbf{E}_{\mathrm{EFI}}$) to that predicted from $\mathbf{E}' = -\mathbf{V \times B}$ allows one to estimate the magnitude and variation of the boom shorting factor, if any.

The effects of spin-dependent current collection, as well as any large-scale space-charge asymmetries surrounding the spacecraft and its appendages (spacecraft photoelectron cloud; electrostatic structures (wakes) arising from ambient cold plasma interactions; etc.) will also show up in this sort of cross-calibration analysis, allowing for estimation of their magnitude and impact on plasma flow and EM fluctuation measurements, as well as optimization of sensor bias settings to reduce those magnitudes, if necessary. This will include the transients that arise when a sensor enters the solar or magnetic shadow of the spacecraft. Either sort of shadowing can occur when the Sun-Earth line or the local magnetic field is within 10 degrees of the SC spin plane, which may occur in the ROI for solar shadows, and in regions near the southern cusp and nightside northern mid-latitudes for magnetic shadows. Such transients are typically easy to spot, and arise from the change in sensor floating potential that occurs when some of the charged particle fluxes to the sensor are changed abruptly (interruption of photoelectron emission, or blockage of B-field-aligned fluxes by the SC body).

In order to make accurate estimates of these calibration parameters and their uncertainties, the cross-calibration process on TRACERS will use well-established statistical methods of on-orbit calibration to make those estimates (Andre et al. 2021; Lejosne and Mozer 2019; Lejosne et al. 2022), and will proceed much like the routine on-orbit calibration of science-grade magnetometers in LEO (Broadfoot et al. 2022). The strong and predictable background electric field arising from the motion of TRACERS across the Earth’s magnetic field provides a zero-order field with which to cross-calibrate the measured potential differences between EFI sensors. This allows for the determination of any slowly varying (multi-spin to orbit-period) biases in the attitude and ephemeris data, and incorporation of those corrections into the estimate of geophysical fields into an Earth-fixed, co-rotating frame.

The magnetic field at TRACERS also includes geophysical perturbations that can be up to 100 nT in magnitude (e.g. the geomagnetic perturbations, $\boldsymbol{\delta} \mathbf{B}$ due to ionospheric or magnetospheric current systems (e.g. the auroral electrojet and field-aligned currents; the Sq current system at mid- and equatorial latitudes, and the Dst due to ring current enhancements during geomagnetic storms). Such perturbations, when combined with the SC velocity, can amount to a small relative perturbation ($<4 \cdot 10^{-3}$) but a measurable (0.8 mV/m) absolute perturbation, the effect of which will be discussed below.

Ground modeling and testing of the GNC systems performance during static and dynamic (delta-V and re-orientation maneuvers) show that the uncertainties in the SC spin axis pointing and spin phase are no greater than $\pm 0.055$ and $\pm 0.07$ deg ($\pm 0.96$ and $\pm 1.4$ mrad) respectively, equivalent to an uncertainty in any element of $\mathrm{R}_{SC}$ of less than the uncertainty measured in mrad, or $0.1\%$, consuming a very small portion of the allowed error budget (Omar and Reynolds 2024).

Similarly, the meter-scale and cm/s-scale uncertainties in modern GPS-based ephemeris solutions amount to relative errors in $\mathrm{V}$ or in model $\mathrm{B}$ on the order of $1\cdot 10^{-5}$, and thus consume an insignificant amount of the allowed error budget.

In addition to these systematic errors in the zero-order model field $\mathbf{E}'$ that arise from the uncertainties in $\mathbf{B}$, $\mathbf{V}$, or $\mathbf{R}_{SC}$, there can be significant geophysical perturbations to the model field $\mathbf{E}'$ in certain “active” regions of the TRACERS orbit, e.g. in the auroral oval or in association with equatorial Spread-F. These perturbations arise from ion flows or geomagnetic perturbations ($\mathbf{E}$ and $-\mathbf{V}\times \boldsymbol{\delta} \mathbf{B}$) in the Earth-fixed frame, and can amount to 1-100 mV/m from $\mathbf{E}$ and up to 0.8 mV/m from $-\mathbf{V}\times \boldsymbol{\delta} \mathbf{B}$. For TRACERS we will use the standard practice of initially excluding such active regions from the spin-by-spin calibration process (automatically, using the outlier removal described below), and then interpolating the calibration parameters from the surrounding “quiet” regions to get the results applicable to the active regions (Broadfoot et al. 2022; Elphic et al. 2001).

In order to determine the quasi-static calibration parameters (offsets, shorting factors, alignment angles) from the $\mathbf{E}'$ estimate we use the data spin by spin from multiple spins worth of both pairs of opposing sensors (every 6 seconds at the nominal 10 RPM spin rate, for 768 pts/spin in the ROI and 48 pts/spin in BOR per axis), fitted to offset sinusoids to determine the shorting factor, angular offset, and DC offset of each pair during that spin.

These factors are expected to vary slowly relative to spin rate as the illumination, ambient plasma conditions, and motion relative to the local B field changes over the orbit, and so spin-by-spin fitting is an appropriate method to estimate them. Any significant outliers in the voltage differences that arise from shadowing, wakes, or geophysical perturbations are dropped from the fitting procedure using an iterative outlier identification and removal algorithm similar to those used on past missions (Polar, THEMIS, RBSP-EFW), and consistent with relevant statistical procedures (Tibshirani 1996). All the data is kept on ground for detailed interpretation so that we will be able to characterize any persistent sub-spin uncertainties as well.

As discussed in Sect. 3, and has been done on past missions, we then use these spin-fit calibration parameters to convert the sample-by-sample potential differences from the two antenna pairs to the fully 2D $\mathrm{E}'$ field estimate in the TRACERS spin plane, and then the fully 3D $\mathbf{E_{\perp}}$ using $\mathbf{E \times B} = 0$ to determine the unmeasured axial component of $\mathbf{E_{\perp}}$.

## Summary

The TRACERS-EFI provides high-quality estimates of the perpendicular components of the DC electric field, allowing for accurate (better than 3 mV/m) estimation of the DC perpendicular components of $\mathbf{E}$ in the relevant plasma environments to support the TRACERS plasma flow observations in the ROI. 2D E field observations up to 1 kHz allow for the identification of electromagnetic fluctuations and estimation of their impact on local particle distributions. 1D E field snapshot observations at frequencies up to 10 MHz provide for the accurate estimate of local plasma conditions and aid in the interpretation of lower-frequency EM fluctuation data.

The EFI team looks forward to sharing our data and results with the community, both in terms of the primary science objectives of the TRACERS mission, and in terms of the collaborative science that can be done between TRACERS and the rest of the Heliophysics community, from the ionosphere out to the magnetosphere and beyond.

## Appendix A: An Electrostatic Cleanliness Specification (ESC) for the TRACERS Mission

The accurate measurement of ambient electric fields in a plasma environment requires careful attention to and control of differential charging of the host spacecraft. An electrostatic cleanliness (ESC) specification defines the design, implementation, and verification process for achieving electrostatic cleanliness as derived from the required accuracy of the electric field measurements and the expected operational environment of the mission. Here, we detail how the TRACERS ESC specification was derived and implemented, and point out the differences found between an ESC specification applicable to LEO spacecraft as opposed to magnetospheric spacecraft.

The understanding that careful consideration of internal and surface charging effects, and mitigation of possible sources of strong differential charging in tenuous plasma conditions is part of robust spacecraft design has deepened over the past two decades (Garret and Whittlesey 2012). These studies have focused on physical damage to electronics and operational anomalies due to total dose, single-event, and differential internal charging effects. However, the TRACERS science objectives for DC E-field and low-energy plasma measurements impose more stringent requirements on the magnitude of charging of spacecraft surfaces (a few volts to a few tens of volts) as well as differential charging between those surfaces (<10 V) than is typical for low-Earth, geosynchronous, or high-Earth orbit spacecraft (LEO, GEO, and HEO, respectively) that are not required to make such measurements.

Realization of this fact led to an early and vigorous effort on the part of the TRACERS instrument and spacecraft engineering teams towards providing an electrostatically clean spacecraft. This effort brought together the EFI science and engineering teams, and the lead mechanical, electrical, thermal, and systems engineers from the instrument and spacecraft teams in order to do the following: specify the requirements for ESC on TRACERS; determine any problem areas on the spacecraft early and decide what, if any forms of mitigation could be used; select and verify appropriate materials and designs for exposed surfaces and the grounding thereof; and finally validate the final ESC implementation throughout the Instrument Suite and Spacecraft I&T flows.

Although stringent ESC programs have been imposed on previous large-scale missions (FAST, Polar, Cluster-II, etc.), the aggressive schedule and limited budget of TRACERS meant that the implementation had to be streamlined and relatively foolproof in order to avoid both costs incurred to develop the implementation (large-scale electrostatic modeling, for example), as well as to ensure that the risk of finding an ESC issue late in the I&T flow was minimized. As was done successfully on earlier NASA missions (Bonnell et al. 2008), this goal of the management process led to the development of a specification for the design, implementation, and verification of an electrostatically clean SC. This specification was captured in the TRACERS Electrical System Specification (ESS). It consisted of a modest library of surfaces and grounding schemes (round and square patches, grounded at corners or edges; surfaces embedded in apertures; insulating epoxy bond lines; etc.) that allowed for the specification of maximum levels of surface resistivity (for materials selection), as well as determination of the maximum allowed resistance to ground from any point on such a surface once installed (allowing for simple verification using an ohmmeter, rather than a surface resistance measurement). This is in contrast to the often used specification of a global maximum allowed surface resistivity for all surfaces on a given spacecraft, which tends to be stricter (and thus harder to comply with) as it does not take into account the inverse relationship between allowed maximum surface resistance and exposed area of any given sub-assembly.

For the TRACERS environment this relationship was that the allowed resistance to ground of any exposed surface was at most 20 M$\Omega \cdot \mathrm{cm}^{2}/\mathrm{A}$, where $\mathrm{A}$ was the exposed area in $\mathrm{cm}^{2}$, under the assumption that the maximum differential current collection/emission was $40~\mathrm{nA}/\mathrm{cm}^{2}$ between any two exposed surfaces (e.g. ram and wake-facing surfaces). For reference, the dominant current density to the SC ambient comes from the thermal electron flux at $8.5~\mathrm{nA}/\mathrm{cm}^{2}$ ($n_{e} = 10^{4}~\mathrm{1}/ \mathrm{cm}^{3}$, $T_{e} = 0.1~\mathrm{eV}$), with ram ion fluxes and photoelectron emission fluxes of 1.2 and $4~\mathrm{nA}/\mathrm{cm}^{2}$ respectively.

When adapting the TRACERS ESC specification from past instances used on the magnetospheric missions listed above, we had to take into account the significant differences between the magnetospheric and LEO environments. There are many differences between these environments, but the most significant are as follows:

- The colder, denser LEO plasma environment ($n_{e} = 10^{3}$ to $10^{6}~\mathrm{1}/\mathrm{cm}^{3}$, $T_{e} \approx T_{i} = 0.1$ to $0.5~\mathrm{eV}$) experienced by TRACERS differs hugely from that experienced in the magnetosphere ($n_{e} = 0.1$ to $1~\mathrm{1}/\mathrm{cm}^{3}$, $T_{e} \leq T_{i} = 10~\mathrm{eV}$ to tens of $\mathrm{keV}$) As noted in the discussion regarding maximum differential current collection above, this plasma environment leads to ram and thermal current densities on the order of or significantly higher than photoelectron current densities in LEO, the opposite of that experienced in the magnetosphere under sunlit conditions. It also leads to much shorter Debye lengths, $\lambda _{De}$, on the order of a cm rather than tens of meters - resulting in much more efficient shielding of differentially charged areas, and thus more allowance for non-compliance when considering exposed insulators such as the patch antennas discussed below.
- The density of Atomic Oxygen (AO), neutral or ionized, at TRACERS altitudes, and TRACERS orbital speed leads to a significant flux of energetic AO ($>4~\mathrm{eV}$) upon all exposed surfaces, SC or instrument. Long-duration orbital exposure studies, as well recent laboratory studies and flight performance of double probe E-field sensors LEO have demonstrated that this energetic AO flux erodes or chemically converts the exposed surfaces of SC and instruments, leading to significant changes and decay of the mechanical, thermal, photoemissive, and conductive properties of those materials, and that one has to take care in the selection and application of standard probe and SC materials for magnetospheric applications in the LEO environment (Visentine et al. 1985; Bonnell and Lanzerotti 2015; Samaniego et al. 2018, 2019; Pfaff et al. 2021).
  For TRACERS these AO effects played a role in two major design choices: First, the choice to apply 11 times the usual thickness of DAG-213 to the EFI sensor surfaces and 3 times the usual thickness to the EFI stacer booms. Second, to accept the application of a dendritic (i.e. non-continuous) insulative Mg-F anti-reflection layer on top of the usual conductive Indium-Tin Oxide (ITO) applied to the solar array cover glasses that serves to improve the solar array photo-conversion performance, and to protect the AO-sensitive (erosion) ITO from direct AO flux.
- The trans- or supersonic passage of TRACERS through the plasma leads to the formation of an electrostatic plasma wake along or across the local magnetic field. This is one of the strongest sources of differential current collection in the TRACERS environment, with the fluxes from the wake expected to be a small fraction of those experienced on ram surfaces. Based on models and observations of the lunar wake (Farrell et al. 1998), the short Debye lengths in LEO will serve to contain the non-geophysical potential perturbations to a “Mach cone” behind the SC. This should limit the impact of the wake on the double probe E-field measurements that has been observed in the magnetospheric and solar wind contexts (Andre et al. 2021), and the on-orbit cross-calibration procedure described in Sect. 4.2 will allow us to estimate and remove its effects.

Verification of proper surface resistance and grounding was performed as early as possible in the integration and test flow, and was an integral part of the closeout and quality assurance process of each instrument, spacecraft subsystem, and completed vehicle. Specialized tools and techniques were developed and tested for use on potentially fragile surfaces, such as the Mg-F, Indium-Tin-Oxide-coated and Germanium Black Kapton surfaces of certain thermal tapes and coatings, in order to provide a robust measurement of the resistances involved (a few k$\Omega $ to tens of M$\Omega $) while allowing a broad range of personnel to use the equipment (thermal treatment technicians, I&T engineering staff, even instrument scientists).

As examples of the TRACERS ESC specification in action, we summarize two of the several activities performed by the TRACERS team to ensure the electrostatic cleanliness required for mission success.

The first example of the use of the ESC was the early identification of some unavoidable exposed insulators on the surface of the spacecraft, and the evaluation of their impact on the EFI measurements. These exposed insulators consisted of the insulative polymer surface of the GPS and S-band telemetry patch antennas (+ and −Z decks), the glass cover for the miniature spinning sun sensor (side panel), and the hard-anodized Aluminum finishes on the Motorized Lightband (−Z deck). The exposed insulative surfaces on these items were key to their function, and could not be swapped for dissipative or conductive materials without seriously compromising their function, incurring significant material and schedule costs, or both. These items were identified by the relevant subsystem leads at the TRACERS SC vendor, Millennium Space Systems (MSS, El Segundo, CA, USA), reported to the relevant system engineering personnel within the TRACERS Project (system engineers and the EFI instrument lead), and their locations and dimensions evaluated as to their impact on systematic offsets to the EFI double probe measurements.

In each case, the exposed insulative area was a few cm in each dimension and was more than three meters away from the nearest EFI sensor surface. This small size (on the order of a few Debye lengths), the extent of the grounded dissipative or conductive surfaces that surrounded them (on the order of or larger than the patch dimensions), and their distance from the EFI sensors (tens of Debye lengths), meant that a simple shielded monopole model of the potential surrounding the area could be used. Under such a model, the potential surrounding the charged area falls off with distance like that surrounding a charged sphere, $V(r) = V(a)\exp{[-(r-a)/\lambda _{e}]}\cdot (a/r)$, where $a$ is a characteristic dimension of the patch, $\lambda _{e}$ is the Debye length, $V(a)$ is the differential voltage between the patch and surrounding grounded area, and $r$ the radial distance from the patch to the nearest EFI sensor ($>3~\mathrm{m}$). Given the thermal and drift energies of the ambient plasma and photoelectron populations (a few eV at most), $V(a)$ can be no more than a few volts. This, combined with the very small value of the exponential term, $\exp{(-300)}$, means that these surfaces will not perturb the potentials of the EFI sensors, and could be accepted as part of the design as-is.

The second example of the use of the ESC specification was the verification in 2024 of the flight solar panel surface conductivity and grounding performed by Spectrolab, the TRACERS SC solar array vendor in conjunction with MSS and other TRACERS personnel. As part of the ESC verification after the assembly and test of the flight solar arrays, the resistance from a distal point on each of the solar array cover glasses to the grounding connection for the solar array surface was measured using a high-input-impedance digital multi-meter (DMM), a standard metallic contact to the ground point, and a “soft-touch” DMM probe for contact to the cover glass surface. This soft-touch probe utilized a rounded conductive polymer cover over a metallic body to allow direct electrical contact with the MG-F/ITO-coated surface of the cover glasses with a minimum of risk to the mechanical integrity of the coating.

Using this test equipment and setup, Spectrolab found that a small percentage of the coverglass resistance measurements consistently failed with too high a resistance to ground while the rest consistently passed: 25 failed out of 721 coverglass measurements, 3 to 4% of the ≈ 18,500 $\mathrm{cm}^{2}$ coverglass area. This reduction in the current collection area of the SC does not significantly impact the capability of the EFI sensor biasing system. Spectralab also found that the largest contiguous patch of failed coverglasses was 5 cells for a total area of 120 $\mathrm{cm}^{2}$. Much like the exposed insulative patches discussed above, this size of a patch does not represent a risk to the EFI measurement. Given those results, the flight solar arrays were accepted as-is for flight.

The ESC specification was applied similarly in many other cases across the spacecraft and instruments, with instrument and spacecraft engineering staff bringing important or suspect design elements to the attention of the ESC review board for discussion, analysis, and disposition. This organic and enthusiastic support for its use across the TRACERS project demonstrates the value of establishing a straightforward and easy to implement specification, of candidly discussing its importance with regard to the EFI measurements with the entire project team, and of explicating the physical rationale behind the design philosophy in ways that make sense across all the disciplines involved. The true test of the specification’s success lies in the EFI measurements on orbit, but its basis in past successful efforts and the consistent application of it provides confidence that it will provide the required environment for EFI to make the required measurements on TRACERS.

## Appendix B: General Abbreviations Used in This Paper

While the special abbreviations used in this paper have been defined as they arose in the text, there are a variety of abbreviations used routinely in the hardware and E-field communities that may not be known to our broader audience. Here, we collect those abbreviations (acronyms, initialisms, etc.) and what they stand for your reference.

AC: alternating current; used generically to refer to a temporally fluctuating field.
DC: direct current; used generically to refer to a steady (or slowly varying) value of a field.
AWG: American Wire Gauge.
ULF: Ultra Low Frequency, traditionally defined in magnetospheric physics as frequencies from a few mHz to between a few tens of Hz to 100 Hz.
ELF: Extremely Low Frequency, traditionally defined in the context of auroral and ionospheric physics as extending from the top of the ULF band up to 3 kHz.
VLF: Very Low Frequency, running from 3-30 kHz.
HF: High Frequency, running from the top of the VLF band up to 10 MHz, and used as a shorthand description for the combination of the LF, MF, and HF bands in the standard designation.
CCSDS: Consultative Committee for Space Data Systems, an international body that established one of the most common packetized data formats used for spacraft telemetry.
SPENVIS: The European Space Agency’s SPace ENVironment Information System, a well-organized set of tools for modeling spacecraft orbits and environments.
GPS: Global Positioning System, the US contribution to the Global Navigation Satellite Systems developed and maintained by various governments that allow precise estimation of position and timing using received signals from constellations of Earth-orbiting satellites.
S-band: The portion of the radio frequency spectrum that extends from 2 to 4 GHz, and that has been used for decades to support satellite command and data communications.

## References

[CR1] Andersson L, Ergun RE, Delory GT, Eriksson A, Westfall J, Reed H, McCauley J, Summers D, Meyers D (2015) The Langmuir Probe and Waves (LPW) instrument for MAVEN. Space Sci Rev 195:173–198. 10.1007/s11214-015-0194-3

[CR2] André M, Eriksson AI, Khotyaintsev YV, Toledo-Redondo S (2021) The spacecraft wake: interference with electric field observations and a possibility to detect cold ions. J Geophys Res Space Phys 10.1029/2021JA029493

[CR3] Bale SD, Goetz K, Harvey PR, Turin P, Bonnell JW, Dudok de Wit T, Ergun RE, MacDowall RJ, Pulupa M, Andre M, Bolton M, Bougeret J-L, Bowen TA, Burgess D, Cattell CA, Chandran BDG, Chaston CC, Chen CHK, Choi MK, Connerney JE, Cranmer S, Diaz-Aguado M, Donakowski W, Drake JF, Farrell WM, Fergeau P, Fermin J, Fischer J, Fox N, Glaser D, Goldstein M, Gordon D, Hanson E, Harris SE, Hayes LM, Hinze JJ, Hollweg JV, Horbury TS, Howard RA, Hoxie V, Jannet G, Karlsson M, Kasper JC, Kellogg PJ, Kien M, Klimchuk JA, Krasnoselskikh VV, Krucker S, Lynch JJ, Maksimovic M, Malaspina DM, Marker S, Martin P, Martinez-Oliveros J, McCauley J, McComas DJ, McDonald T, Meyer-Vernet N, Moncuquet M, Monson SJ, Mozer FS, Murphy SD, Odom J, Oliverson R, Olson J, Parker EN, Pankow D, Phan T, Quataert E, Quinn T, Ruplin SW, Salem C, Seitz D, Sheppard DA, Siy A, Stevens K, Summers D, Szabo A, Timofeeva M, Vaivads A, Velli M, Yehle A, Werthimer D, Wygant JR (2016) The FIELDS instrument suite for Solar Probe Plus: Measuring the coronal plasma and magnetic field, plasma waves and turbulence, and radio signatures of solar transients. Space Sci Rev 204:49–82. 10.1007/s11214-016-0244-5 29755144 10.1007/s11214-016-0244-5 PMC5942226

[CR4] Bonnell J, Lanzerotti LJ (2015) Neutral oxygen effects at low Earth altitudes: a critical uncertainty for spacecraft operations and space weather effects. Space Weather 13:396–397. 10.1002/2015SW001229

[CR5] Bonnell J, Kintner P, Wahlund J-E, Holtet JA (1997) Modulated Langmuir waves: Observations from Freja and SCIFER. J Geophys Res Space Phys 102(A8):17233–17240. 10.1029/97JA01499

[CR6] Bonnell JW, Mozer FS, Delory GT, Hull AJ, Ergun RE, Cully CM, Angelopoulos V, Harvey PR (2008) The Electric Field Instrument (EFI) for THEMIS. Space Sci Rev 141:303–341. 10.1007/s11214-008-9469-2

[CR7] Broadfoot RM, Miles DM, Holley W, Howarth AD (2022) In situ calibration of the Swarm-Echo magnetometers. Geosci Instrum Method Data Syst 11:323–333. 10.5194/gi-11-323-2022

[CR8] Burch JL, Hwang KJ (2021) Exploring small scales with MMS. In: Maggiolo R, et al. (eds) Magnetospheres in the Solar System. Wiley-AGU, Hoboken, pp 657–671. 10.1002/9781119815624.ch41

[CR9] Burch JL, Phan TD (2016) Magnetic reconnection at the dayside magnetopause: advances with MMS. Geophys Res Lett 43:8327–8338. 10.1002/2016GL069787

[CR10] Califf S, Cully CM (2016) Empirical estimates and theoretical predictions of the shorting factor for the THEMIS double-probe electric field instrument. J Geophys Res Space Phys 121:6223–6233. 10.1002/2016JA022589

[CR11] Christopher IW, Kletzing CA, Crawford D, Piker C, Wilkinson D, Steele K, Petrinec SM, Bounds S, Vaclavik S, Omar S, Shults E, Winter M, Miles DM (2025) The Tandem Reconnection and Cusp Electrodynamics Reconnaissance Satellites (TRACERS) science operations center. Space Sci Rev 221. 10.1007/s11214-025-01199-x 10.1007/s11214-025-01199-x PMC12339611 40810049

[CR12] Elphic RC, Means JD, Snare RC, Strangeway RJ, Kepko L, Ergun RE (2001) Magnetic Field Instruments for the Fast Auroral Snapshot Explorer. Space Sci Rev 98:151–168. 10.1023/A:1013153623344

[CR13] Ergun RE, Carlson CW, Mozer FS, Delory GT, Temerin M, McFadden JP, Pankow D, Abiad R, Harvey P, Wilkes R, Primbsch H, Elphic R, Strangeway R, Pfaff R, Cattell CA (2001) The FAST satellite fields instrument. Space Sci Rev 98:67–91. 10.1023/A:1013131708323

[CR14] Farrell WM, Kaiser ML, Steinberg JT, Bale SD (1998) A simple simulation of a plasma void: Applications to Wind observations of the lunar wake. J Geophys Res Space Phys 103(A10):23653–23660. 10.1029/97JA03717

[CR15] Garret HB, Whittlesey AC (2012) Guide to mitigating spacecraft charging effects. Wiley, New York. 10.1002/9781118241400

[CR16] Gustafsson G, Bostrom R, Holback B, Holmgren G, Lundgren A, Stasiewicz K, Ahlen L, Mozer FS, Pankow D, Harvey P, Berg P, Ulrich R, Pedersen A, Schmidt R, Butler A, Fransen AWC, Klinge D, Thomsen M, Falthammar C-G, Lindqvist P-A, Christenson S, Holtet J, Lybekk B, Sten TA, Tanskanen P, Lappalainen K, Wygant J (1997) The electric field and wave experiment for the Cluster mission. Space Sci Rev 79:137–156. 10.1023/A:1004975108657

[CR17] Harvey P, Mozer FS, Pankow D, Wygant J, Maynard NC, Singer H, Sullivan W, Anderson EB, Pfaff R, Aggson T, Pedersen A, Falthammar C-G, Tanskannen E (1993) The electric field instrument on the Polar satellite. Space Sci Rev 71:583–596. 10.1007/BF00751342

[CR18] Heppner JP, Bielecki AE, Aggson TL, Maynard NC (1978) Instrumentation for DC and low-frequency electric-field measurements on ISEE-A. IEEE Trans Geosci Electron GE-16(3):253–257. 10.1109/TGE.1978.294557

[CR19] Hospodarsky GB, Carton AJ, Dvorsky RT, et al. (2025) The Magnetic Search Coil (MSC) on the TRACERS Mission. Space Sci Rev 221:72. 10.1007/s11214-025-01200-7 40777824 10.1007/s11214-025-01200-7 PMC12325441

[CR20] Lejosne S, Mozer FS (2019) Shorting factor in-flight calibration for the Van Allen Probes DC electric field measurements in the Earth’s plasmasphere. Earth Space Sci 6:646–654. 10.1029/2018EA000550 31080847 10.1029/2018EA000550 PMC6510545

[CR21] Lejosne S, Bonnell JW, Wygant JR, Mozer FS (2022) Maximizing the accuracy of double probe electric field measurements near perigee: The case of the Van Allen Probes instruments. J Geophys Res Space Phys 127:e2021JA030099. 10.1029/2021JA030099

[CR22] McAdams KL, LaBelle J, Trimpi ML, Kintner PM, Arnoldy RA (1999) Rocket observations of banded structure in waves near the Langmuir frequency in the auroral ionosphere. J Geophys Res Space Phys 104:28109–28122. 10.1029/1999ja900379

[CR23] Miles DM, Kletzing CA, Fuselier SA, Goodrich KA, Bonnell J, Cao IH, Cairns H, Chen L-J, Christopher IW, Cleveland K, Connor HJ, Crawford D, Dolan J, Dorelli J, Dvorsky R, Finley MG, Friedel R, Halekas JS, Hospodarsky G, Jaynes A, Labelle J, Lin Y, Petrinec SM, Phillips ML, Powers R, Prasad B, Rospos A, Santolik O, Strangeway R, Trattner KH, Washington A (2025) The Tandem Reconnection and Cusp Electrodynamics Reconnaissance Satellites. (TRACERS) Mission. Space Sci Rev 221:6. 10.1007/s11214-025-01184-4 40584404 10.1007/s11214-025-01184-4 PMC12204947

[CR24] Mozer FS (2016) DC and low-frequency double probe electric field measurements in space. J Geophys Res Space Phys 121:10942–10953. 10.1002/2016JA022952

[CR25] Mozer FS, Torbert RB, Fahleson UV, Falthammar CG, Gonfalone A, Pedersen A (1978) Measurements of quasi-static and low-frequency electric fields with spherical double probes on the ISEE-1 spacecraft. IEEE Trans Geosci Electron GE-16(3):258–261. 10.1109/TGE.1978.294558

[CR26] Naval Research Lab (2021) NRLMSIS 2.0. https://ccmc.gsfc.nasa.gov/models/NRLMSIS~00/. Accessed: 14 Feb 2025

[CR27] Omar S, Reynolds R (2024) TRACERS mission and GNC architecture. In: 38th annual small satellite conference, SSC24-II-01. 10.26077/pqqv-1177

[CR28] Patacchini L, Hutchinson IH (2011) Spherical conducting probes in finite Debye length plasmas and E × B fields. Plasma Phys Control Fusion 53. 10.1088/0741-3335/53/2/025005

[CR29] Pedersen A, Mozer F, Gustafson G (1998) Electric field measurements in a tenuous plasma with spherical double probes. In: Pfaff RF, Borovsky JE, Young DT (eds) Measurement techniques in space plasmas. AGU, Washington, pp 1–12. 10.1002/9781118664391.ch1

[CR30] Petrinec S, Kletzing C, Miles D, Fuselier S, Christopher I, Crawford D, Bounds S, Bonnell J, Halekas J, Hospodarsky G, Strangeway R, Lin Y, Trattner K, Labelle J, Øieroset M, Santolik O, Moen J, Oksavik K, Cairns I, Mark D (2025) The Tandem Reconnection and Cusp Electrodynamics Reconnaissance Satellites (TRACERS) Mission Design. Space Sci Rev 221:60. 10.1007/s11214-025-01185-3 40584405 10.1007/s11214-025-01185-3 PMC12204924

[CR31] Pfaff R, Uribe P, Fourre R, Kujawski J, Maynard N, Acuña M, Rowland D, Freudenreich H, Bromund K, Martin S, Liebrecht C, Kramer R, Hunsaker F, Holzworth R, McCarthy M, Farrell W, Klenzing J, Le G, Jacobson A, Houser J, Steigies C, Berthelier JJ (2021) The Vector Electric Field Investigation (VEFI) on the C/NOFS satellite. Space Sci Rev 217:85. 10.1007/s11214-021-00859-y

[CR32] Sahu K, Kniffen S (1998) PPM-98-008, Radiation report on OP-15 (analog devices) (LDC9722A). Technical report, Unisys Corporation—Federal Systems Division

[CR33] Samaniego JI, Wang X, Andersson L, Malaspina D, Ergun RE, Horányi M (2018) Investigation of coatings for Langmuir probes in an Oxygen-rich space environment. J Geophys Res Space Phys 123:6054–6064. 10.1029/2018JA025563

[CR34] Samaniego JI, Wang X, Andersson L, Malaspina D, Ergun RE, Horányi M (2019) Investigation of coatings for Langmuir probes: Effect of surface oxidation on photoemission characteristics. J Geophys Res Space Phys 124 10.1029/2018JA026127

[CR35] Samara M, Labelle J, Cairns IH (2008) Statistics of auroral Langmuir waves. Ann Geophys 26:3885–3895. 10.5194/angeo-26-3885-2008

[CR36] Tibishirani R (1996) Regression shrinkage and selection via the lasso. J R Stat Soc B 58(1):267–288. 10.1111/j.2517-6161.1996.tb02080.x

[CR37] Visentine JT, Leger LJ, Kuminecz JF, Spiker IK (1985) STS-8 atomic oxygen effects experiment. In: AIAA 23rd aerospace sciences meeting, AIAA-85-0415

[CR38] Wygant JR, Bonnell JW, Goetz K, Ergun RE, Mozer SD, Bale FS, Ludlam M, Turin P, Harvey PR, Hochmann R, Harps K, Dalton G, McCauley J, Rachelson W, Gordon D, Donakowski B, Shultz C, Smith C, Diaz-Aguado M, Fischer J, Heavner S, Berg P, Malsapina DM, Bolton MK, Hudson M, Strangeway RJ, Baker DN, Li X, Albert J, Foster JC, Chaston CC, Mann I, Donovan E, Cully CM, Cattell CA, Krasnoselskikh V, Kersten K, Brenneman A, Tao JB (2013) The Electric Field and Waves instruments on the Radiation Belt Storm Probes mission. Space Sci Rev 179:183–220. 10.1007/s11214-013-0013-7

